# CrossCheck: an open-source web tool for high-throughput screen data analysis

**DOI:** 10.1038/s41598-017-05960-3

**Published:** 2017-07-19

**Authors:** Jamil Najafov, Ayaz Najafov

**Affiliations:** 1 Department of Computer Engineering, Faculty of Engineering, Gazi University, Ankara, Turkey; 2 Department of Cell Biology, Harvard Medical School, Boston, USA

## Abstract

Modern high-throughput screening methods allow researchers to generate large datasets that potentially contain important biological information. However, oftentimes, picking relevant hits from such screens and generating testable hypotheses requires training in bioinformatics and the skills to efficiently perform database mining. There are currently no tools available to general public that allow users to cross-reference their screen datasets with published screen datasets. To this end, we developed CrossCheck, an online platform for high-throughput screen data analysis. CrossCheck is a centralized database that allows effortless comparison of the user-entered list of gene symbols with 16,231 published datasets. These datasets include published data from genome-wide RNAi and CRISPR screens, interactome proteomics and phosphoproteomics screens, cancer mutation databases, low-throughput studies of major cell signaling mediators, such as kinases, E3 ubiquitin ligases and phosphatases, and gene ontological information. Moreover, CrossCheck includes a novel database of predicted protein kinase substrates, which was developed using proteome-wide consensus motif searches. CrossCheck dramatically simplifies high-throughput screen data analysis and enables researchers to dig deep into the published literature and streamline data-driven hypothesis generation. CrossCheck is freely accessible as a web-based application at http://proteinguru.com/crosscheck.

## Introduction

**Figure 1:**
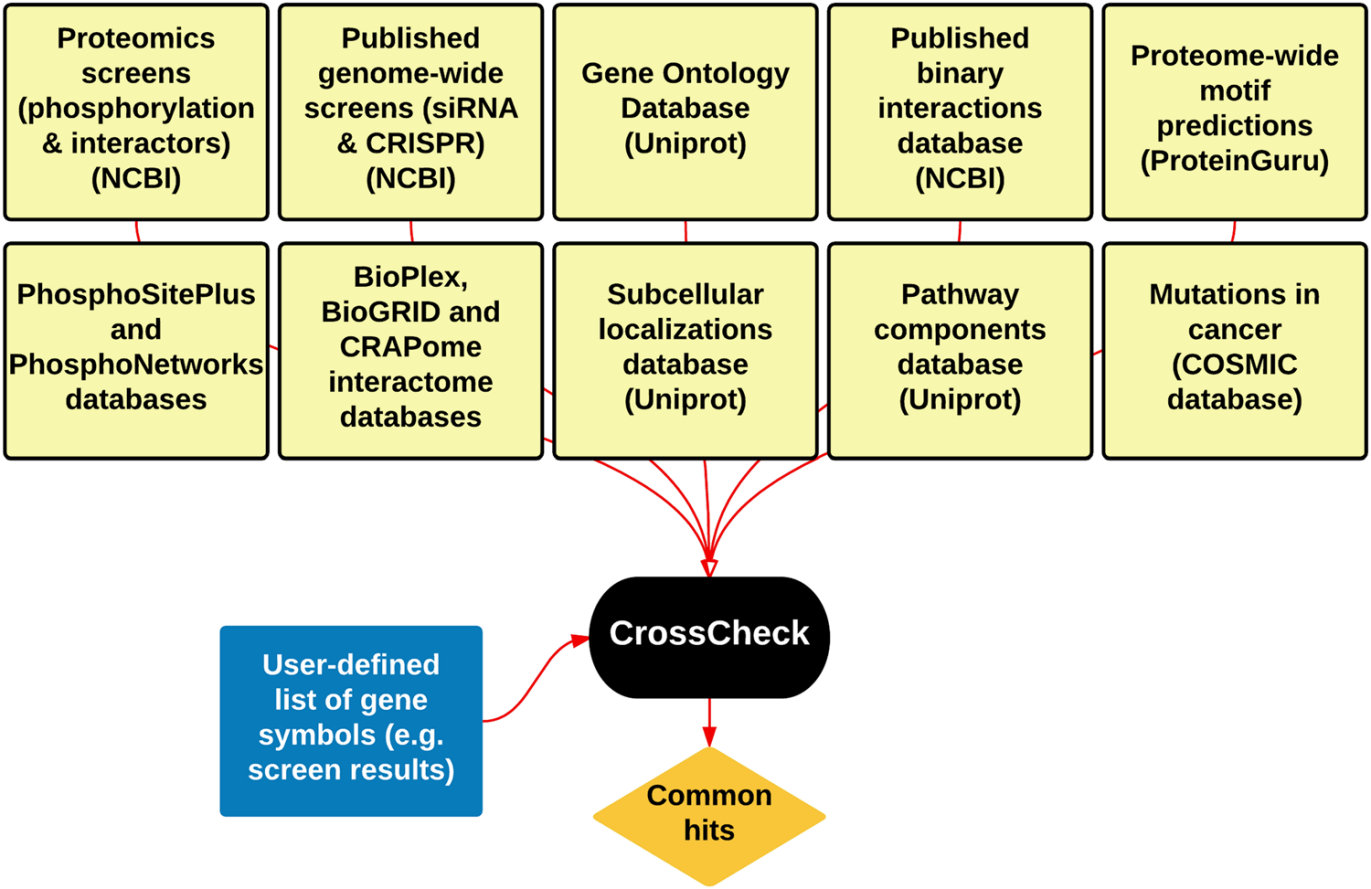
Outline of the CrossCheck reference database sources.

Generation of vast high-throughput datasets has become routine, thanks to recent advances in technologies such as mass spectrometry-based proteomics, genome-wide RNAi and knockout screens followed by deep sequencing, as well as microarray and RNAseq. Productively navigating the landscapes created by such large datasets requires meticulous, complicated and time-consuming database analysis, in order to reveal the useful, biologically-relevant information. It is especially difficult to cross-reference a novel high-throughput screen dataset with the published multitude of high-throughput screen datasets and currently, there are no simple tools for performing such analyses. To address this issue, we developed CrossCheck – a PHP- and JavaScript-based, rapid and user-friendly online software/database that allows comparison of a user-defined list of gene symbols with CrossCheck’s large reference database, which includes published high-throughput screen datasets and low-throughput published information deposited into NCBI databases, as well as a novel predicted protein kinase substrate database (Fig. 1, Supplementary Figures 1 and 2).

## Methods

Algorithms to construct the CrossCheck’s proteome-wide protein kinase substrate motif database, perform the CrossCheck’s cross-referencing function were prepared using PHP. Overview function uses D3.js, a JavaScript data visualization library to map statistics on a heatmap (see http://www.proteinguru.com/crosscheck/codes/). See Supplementary Methods for full description of the methods used.

## Results

### The CrossCheck Reference Database

We have developed a centralized database consisting of manually compiled 16,231 datasets, with 614,161 screen hits, binary interactions and other functional information (Supplementary Table 1). This reference database contains 75 recently-published genome-wide RNAi, CRISPR and insertional knockout screens, as well as proteomics screens, including phosphorylation, ubiquitination and interactome screens. The database also contains known interactors of 1254 metabolic enzymes and various signaling enzymes including 610 kinases, 150 phosphatases and 216 E3 ligases, mined from the NCBI Gene database^1^, as well as gene ontology cluster lists (mined from Uniprot^2^), systematic interactome databases (BioPlex^3^, BioGRID^4^), cancer mutation databases (COSMIC^5^), phosphoproteome databases (PhosphoSitePlus^6^), PhosphoNetworks^7^) and a background/contaminant screen dataset (CRAPOME^8^). By simply pasting and searching a list of gene symbols through CrossCheck, a user with no bioinformatics background or database mining skills is able to cross-reference their dataset across published 16,231 datasets – an unprecedented analytical power heretofore unavailable at any other database or web tool. CrossCheck produces a tab-separated output text file with each column in the output file containing hits common to that dataset and the user-entered dataset. This analysis is done per database selected using the drop-down menu and a separate output file is generated for each analysis. The processing times are outstandingly short, with less than a second for cross-referencing 5355 unique gene symbols with BioGRID v3.4 database that contains 49986 interaction hits for 6981 baits (Supplementary Figure 3).

In contrast to the aforementioned published database tools, which are composed of single type of information, such as protein-protein interaction data or phosphorylation site data, CrossCheck serves as a centralized database and allows rapid analysis across several different types of functional data, such as phenotypic data from genome-wide RNAi/knockout screens, interaction data, protein kinase motif prediction data, gene mutation in cancer data, pathway representation data, and subcellular localization data. With regards to the number of databases or screen datasets incorporated, to our knowledge, no published database is comparable to CrossCheck, as even the largest monotype database, such as BioGRID, currently amounts to only 49986 entries, while CrossCheck currently consists of 614,161 entries. In short, one of the main functionalities of CrossCheck is its service as a multi-faceted centralized database of low- and high-throughput screening datasets that can be effortlessly mined, thereby significantly accelerating high-throughput dataset analysis independent of the database type or experimental source of the data.

### User-Defined Reference Databases

Importantly, in addition to the CrossCheck reference database (Option A, Supplementary Figure 1), users can upload their own reference database (Option B, Supplementary Figure 1) in the tab-separated format (where each column represents a single dataset). Users can therefore intersect any novel high-throughput screen dataset with a multitude of their own unpublished datasets. This important feature gives flexibility and versatility to CrossCheck, allowing users to build their own reference databases and cross-reference them independently of the CrossCheck reference database.

### Proteome-Wide Kinase Substrate Prediction Database

Protein kinases are the cornerstones of cellular signaling cascades and identification of their novel substrates is vital to the progress in understanding the biological functions of kinases and their role in human diseases, as well as how the pathways can be rationally targeted for disease therapies. However, while there are several protein kinase motif databases^9, 10^, no databases with proteome-wide prediction of protein kinase substrates exist. To this end, using reported protein kinase substrates from PhosphoSitePlus^6^, we generated high, medium and low stringency consensus motifs for 347 protein kinases (Supplementary Table 2) and using these motifs, performed a global, proteome-wide kinase substrate prediction (PKSP) search to identify novel putative substrates of these kinases. The resulting search produced 12345, 89432 and 272992 predicted kinase substrates for high, medium and low stringency motifs, respectively. These putative kinase substrates (Supplementary Tables 3, 4 and 5) are deposited into the CrossCheck reference database, allowing users to effortlessly analyze their datasets with an aim of discovering novel kinase targets. Combined with the reported kinase interactors database (also deposited into CrossCheck’s reference database), PKSP database offers an effective prospect to study kinase signaling, since intersection of these databases provides the users with a potent strategy to identify novel protein kinase substrates. As a proof of principle, upon cross-referencing of PKSP with published Akt interactors, DAB2IP was revealed as a predicted Akt substrate (not reported in the PhosphoSitePlus database), and in fact it was reported as a *de facto* Akt substrate in a recent study^11^. In a nutshell, we believe that CrossCheck’s PKSP database will strongly facilitate discovery of novel protein kinase substrates and accelerate cell signaling research.

### The Overview Function

In addition to the cross-referencing function, CrossCheck has an auxiliary Overview function. This heatmap-generating function provides a bird’s-eye preview of the overlap of the user-entered dataset and all the databases in CrossCheck, *en bloc*. In other words, the Overview function will cross-reference a user-entered list of genes with all of the CrossCheck’s databases and produce a heatmap summarizing the abundance of common hits found between the entered dataset and all the databases. Therefore, using this summary, it is very easy to identify which databases in CrossCheck are most relevant to the user’s dataset and perform selective analysis of those databases using the main cross-referencing function of CrossCheck (Supplementary Figures 4–9). The heatmap can be organized via sorting options by gene, by database, and by both. The heatmap is interactive and is bicolor threshold-regulated, in order to allow for simplified visualization of complex datasets. The heatmap also provides common total hit abundance information, as well as relative normalized hit abundance information, abundance scoring by percentage of total database hits and Z-score analysis upon mouse-over of the heatmap cells.

### CrossCheck and Large Datasets

To illustrate the high-throughput capability of CrossCheck, we performed analysis of a published dataset - genome-wide CRISPR screen for essential genes in the human genome^12^. The analyzed dataset for KBM7 cells consists of 2306 gene symbol entries and was cross-referenced with the CrossCheck’s “Genome-wide RNAi and CRISPR Screens” reference database that currently consists of 50 screens. The analysis was completed online in ~2 seconds and produced an output file with 9411 common hits found in 49 out of the 50 screens. The number of discovered common hits ranged from 1 to 1297 per screen, with a mean of 142 and a median of 48 common hits per screen across all screens (Supplementary Figure 10). Importantly, this analysis revealed that 122 out of 2306 essential genes for KBM7 cells are genes mediating TNFα-induced NF-κB pathway activity. Moreover, only 36 of the essential genes were identified as NF-κB pathway targets, suggesting a complex regulation of a focused transcriptional pathway output paradigm. When these 122 hits were re-run through the same database, CASP4 and UBE2M were revealed as the only essential genes that are both mediators of TNFα-induced NF-κB signaling and transcriptional targets of this pathway. Moreover, in the same output file, CASP4 was also found as a modulator of cell death induced by Bortezomib and a host factor affecting *C. burnetii* growth. In short, CrossCheck’s ability to process vast quantities of high-throughput screen data can be a powerful tool for rapid, data-driven hypothesis generation.

### Final remarks

CrossCheck’s reference database is updated quarterly and constantly growing, as new high-throughput screen datasets are published. As is evident from the open-source codes, datasets analyzed through CrossCheck are never copied or stored, therefore providing complete confidentiality of the users’ unpublished data. On the other hand, researchers are encouraged to submit their published datasets to the CrossCheck reference database system by submitting tab-separated files via email to info@proteinguru.com. CrossCheck does not require any training in bioinformatics or user account registration, and is available online for free at http://www.proteinguru.com/crosscheck/. The source code files for the CrossCheck program are open to public at http://www.proteinguru.com/crosscheck/codes/.

In summary, CrossCheck is a powerful centralized database cross-referencing tool, with a potent scope and magnitude of exploration, serving as a versatile data-driven hypothesis generation resource for both low- and high-throughput dataset analysis.

## Electronic supplementary material

Supplementary Methods, Figures and Table 2

Supplementary Table 1

Supplementary Table 3

Supplementary Table 4

Supplementary Table 5
