# Supplementary material for: CrossCheck: an open-source web tool for high-throughput screen data analysis
Source: Sci Rep. 2017 Jul 19;7:5855. doi: 10.1038/s41598-017-05960-3 (PMC5517520; doi:10.1038/s41598-017-05960-3)
Supplement: Supplementary file 1 — Supplementary Methods, Figures and Table 2 [file 41598_2017_5960_MOESM1_ESM.pdf]

CrossCheck: an open-source web tool for high-throughput screen data analysis

Jamil Najafov[1] and Ayaz Najafov[2*]
[1] Department of Computer Engineering, Faculty of Engineering, Gazi University, Ankara, Turkey.
[2]Department of Cell Biology, Harvard Medical School, Boston, USA.
*Correspondence to: ayaz_najafov@hms.harvard.edu

Supplementary Methods

CrossCheck is compatible with all contemporary internet browsers and does not require installation of any additional plug-ins or extensions. Overview visualization function was extensively tested in the latest versions of Chrome and Firefox browsers. There are no limitations incurred by CrossCheck to the size of uploaded dataset or reference database, all resources of hosting enviroment can be used on demand. However, for frequent processing of large datasets (over 20,000 gene symbols and 10MB reference database) it's advised to download and install CrossCheck on a local server.

Generation of the CrossCheck reference database

Database was manually compiled and organized in series of files in tab-separated values format to facilitate viewing and editing in spreadsheet applications as well as in plain text editors.

Generation of protein kinase consensus motifs for proteome-wide predictions

The PhosphoSitePlus substrate phosphorylation database was parsed and motifs were constructed for each kinase as follows. For each position in the motifs consisting of either 12 (low strongency) or 15 (high stringency) residues, consensus coefficient (q) was calculated for each motif position using frequency of appearance of each amino acid in that position (freq) normalized to total number of *different* amino acids appearing at that position. In other words, for each position (*i*) the following formula was used for each position in the motif, where n is the total length of the motif:

$$q_i = \frac{freq(i)}{\sum_{i=0}^{n}(unique\ amino\ acids)}$$

Amino acids were recorded into the motif only when q > 1.2

Supplementary Figure 1

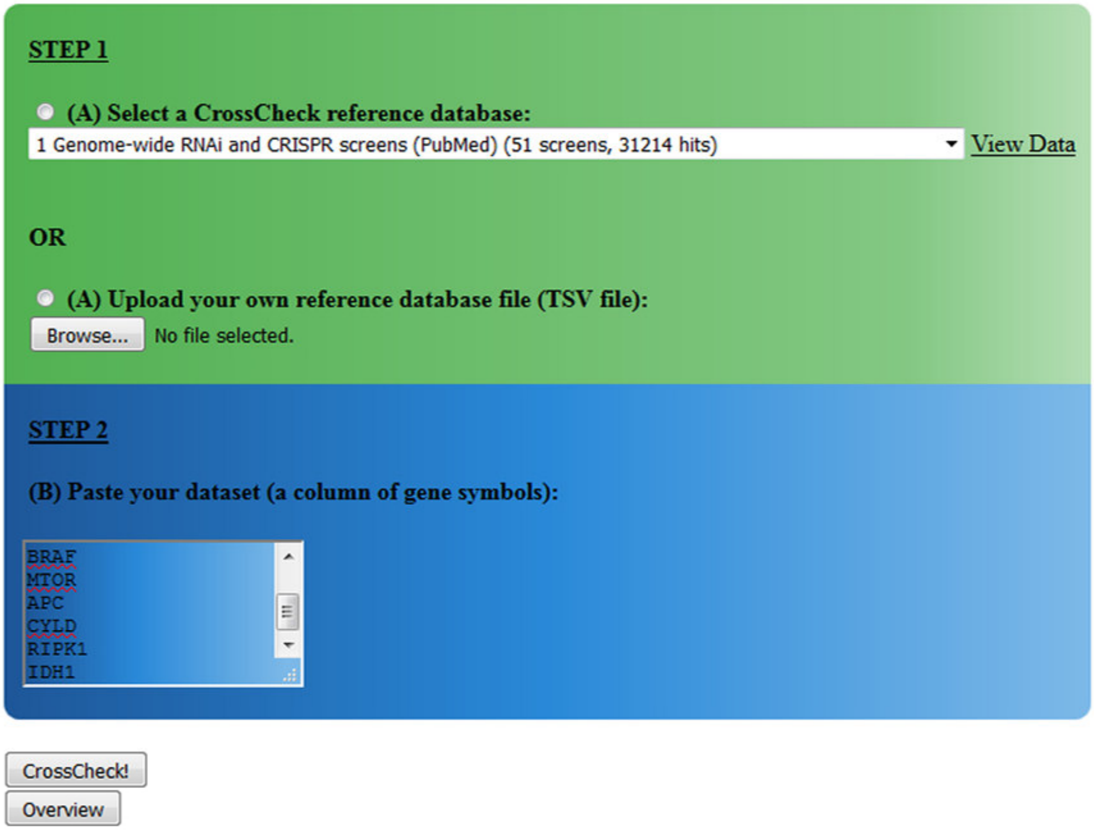

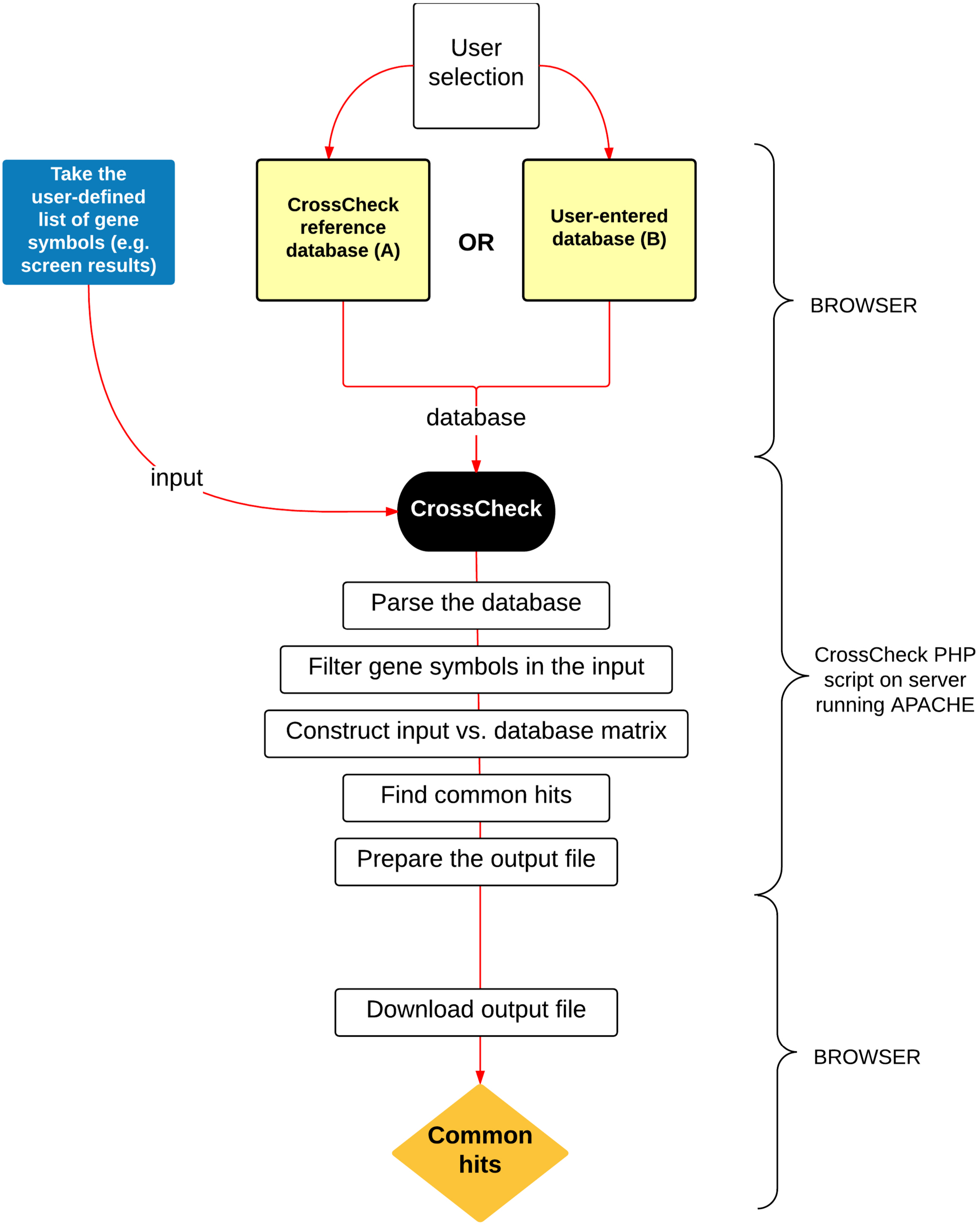
User selection
Take the user-defined list of gene symbols (e.g. screen results)
CrossCheck reference database (A)
OR
User-entered database (B)
BROWSER
database
input
CrossCheck
Parse the database
Filter gene symbols in the input
Construct input vs. database matrix
Find common hits
Prepare the output file
CrossCheck PHP script on server running APACHE
Download output file
BROWSER
Common hits

Supplementary Figure 3

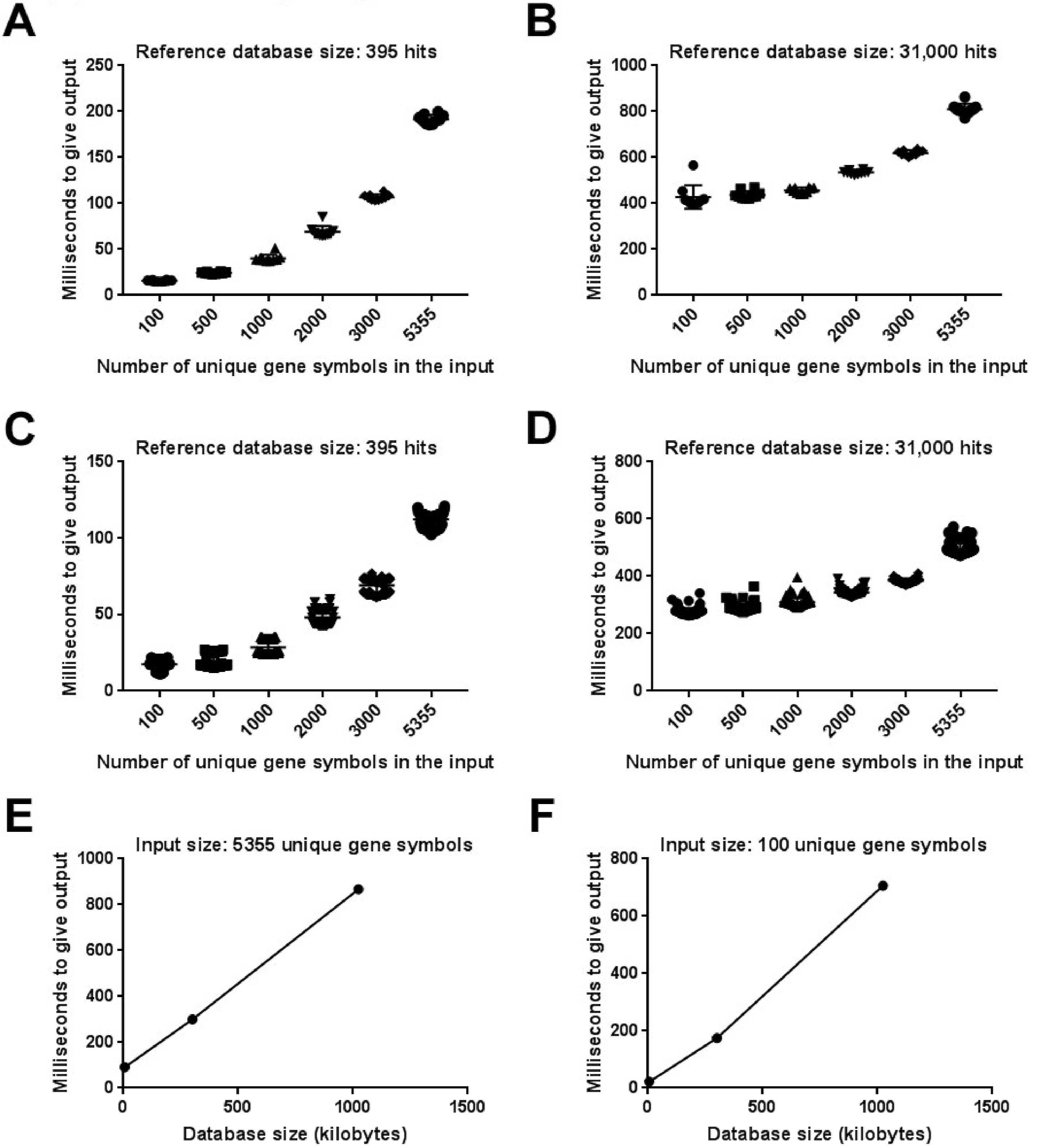

Supplementary Figure 4

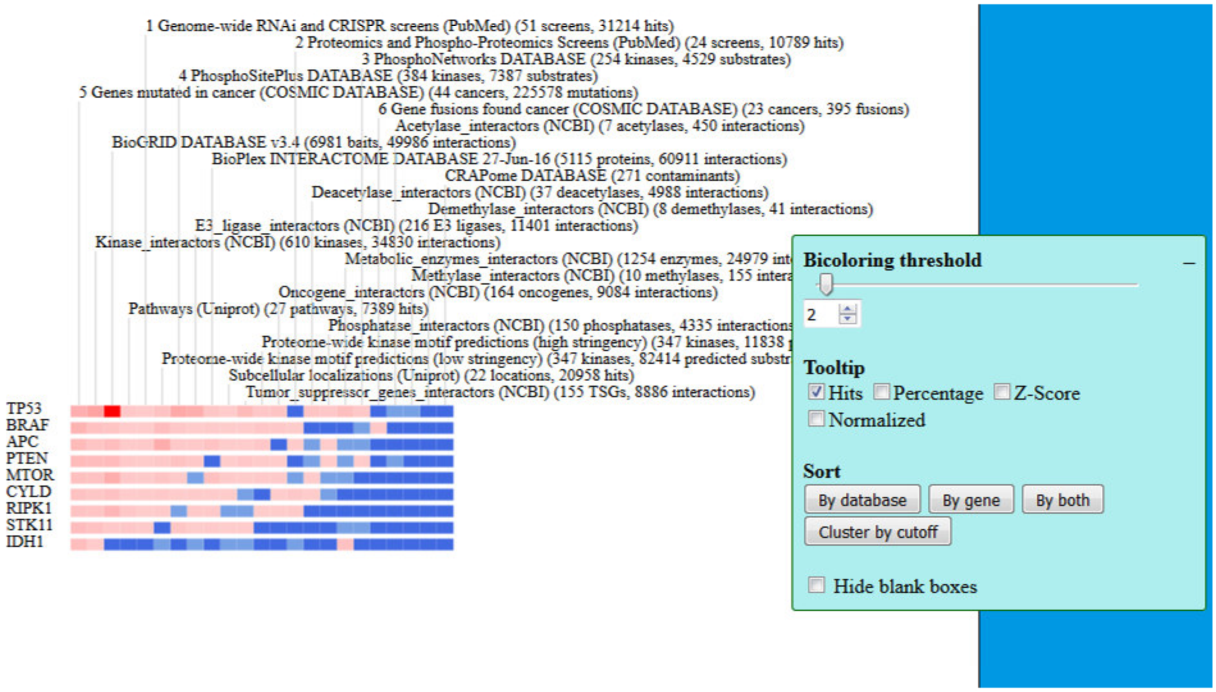

Supplementary Figure 5

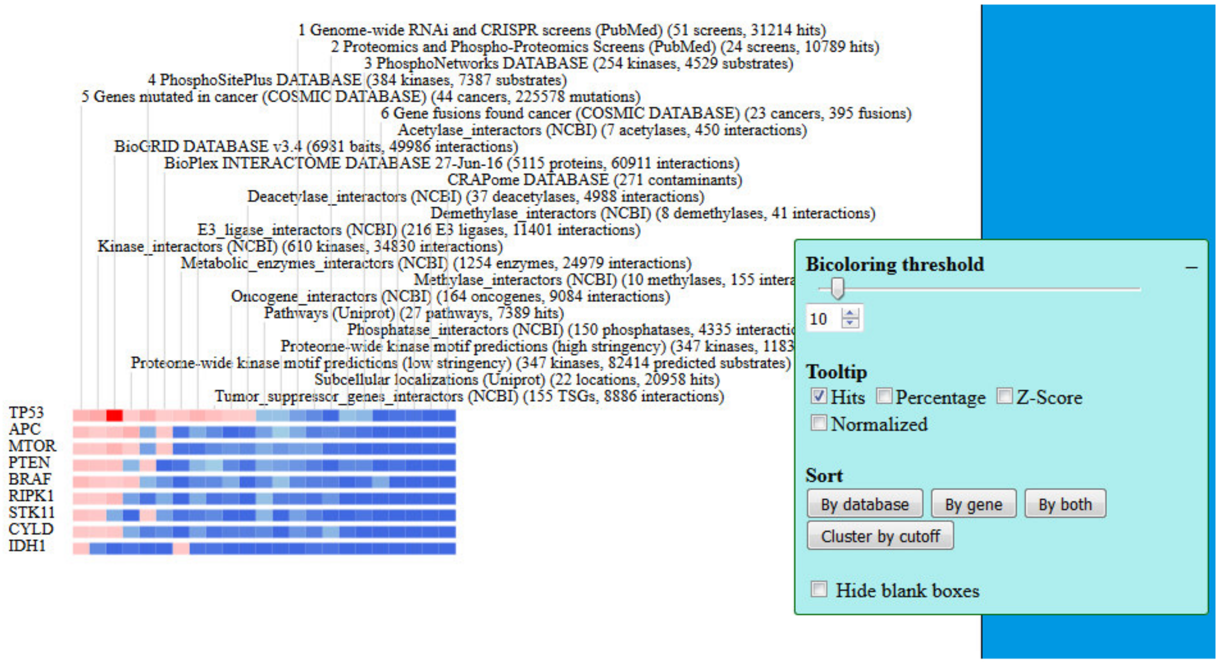

Supplementary Figure 6

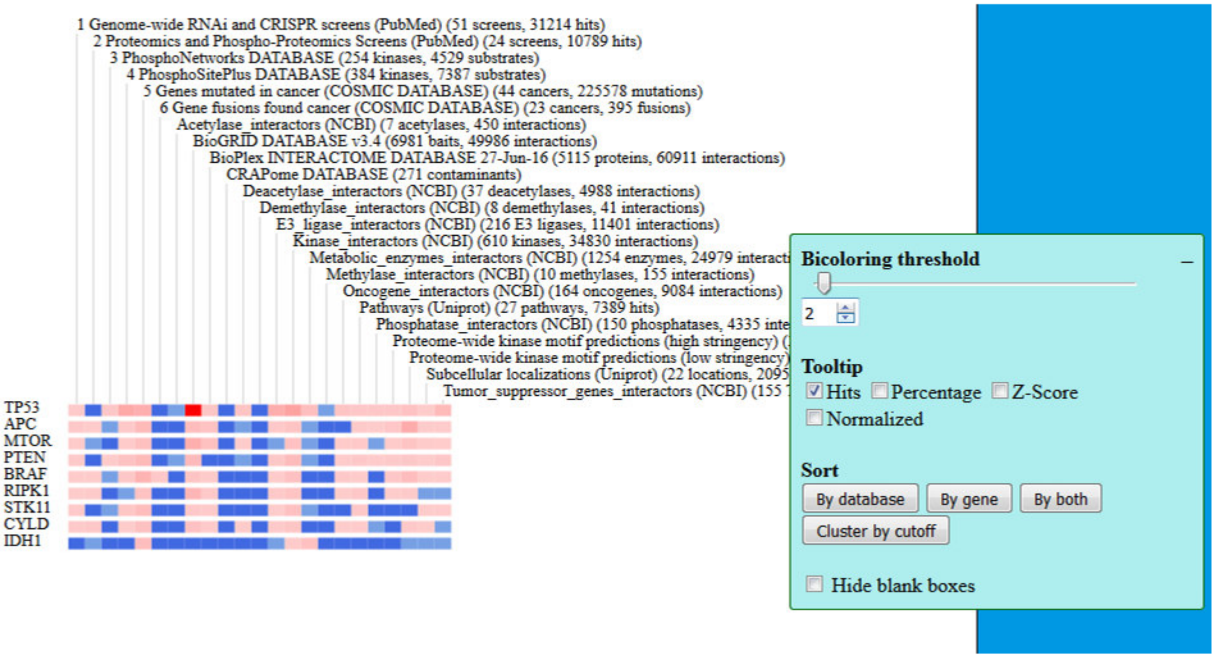

Supplementary Figure 7

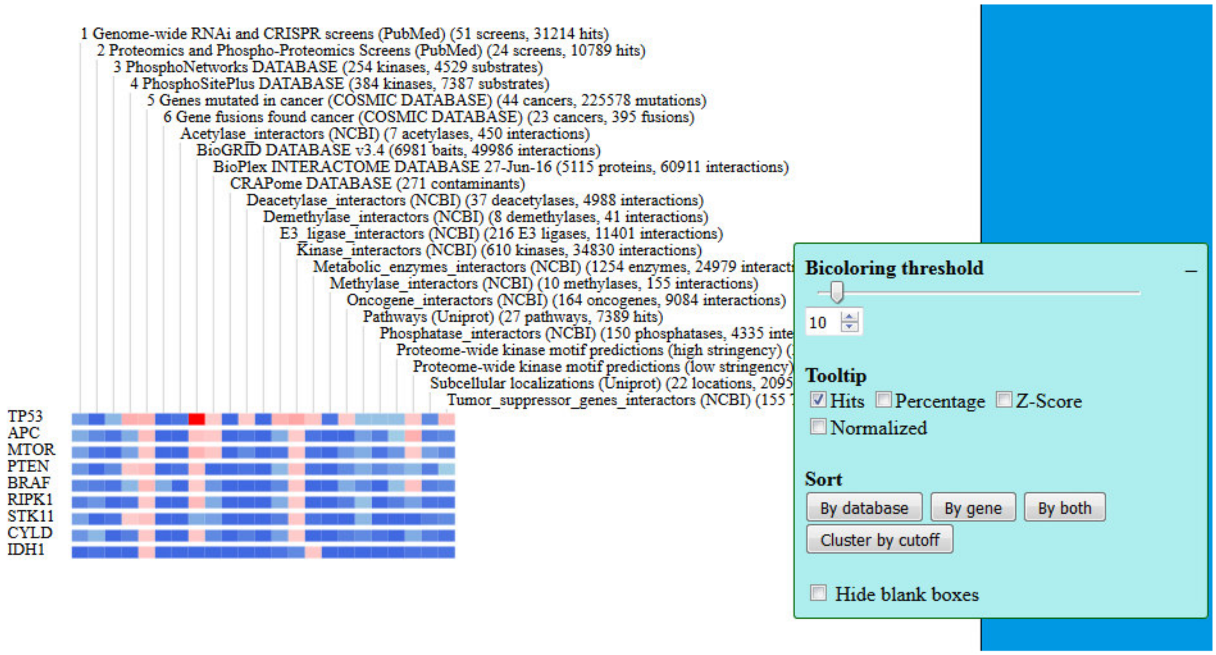

Supplementary Figure 8

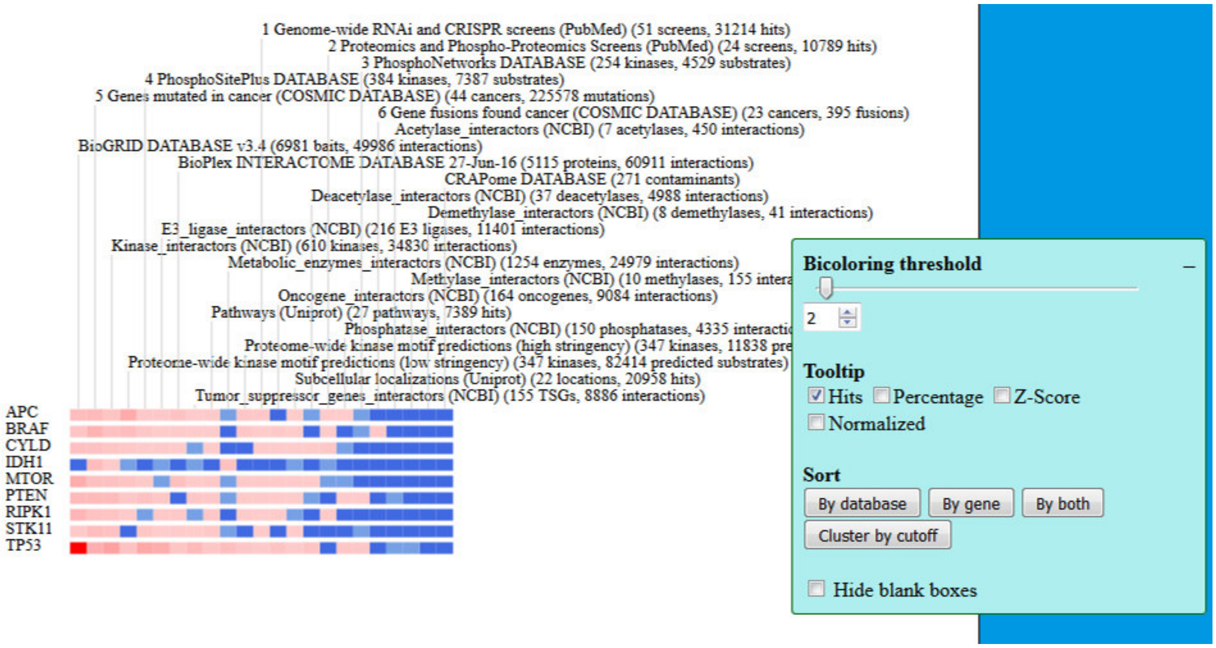

Supplementary Figure 9

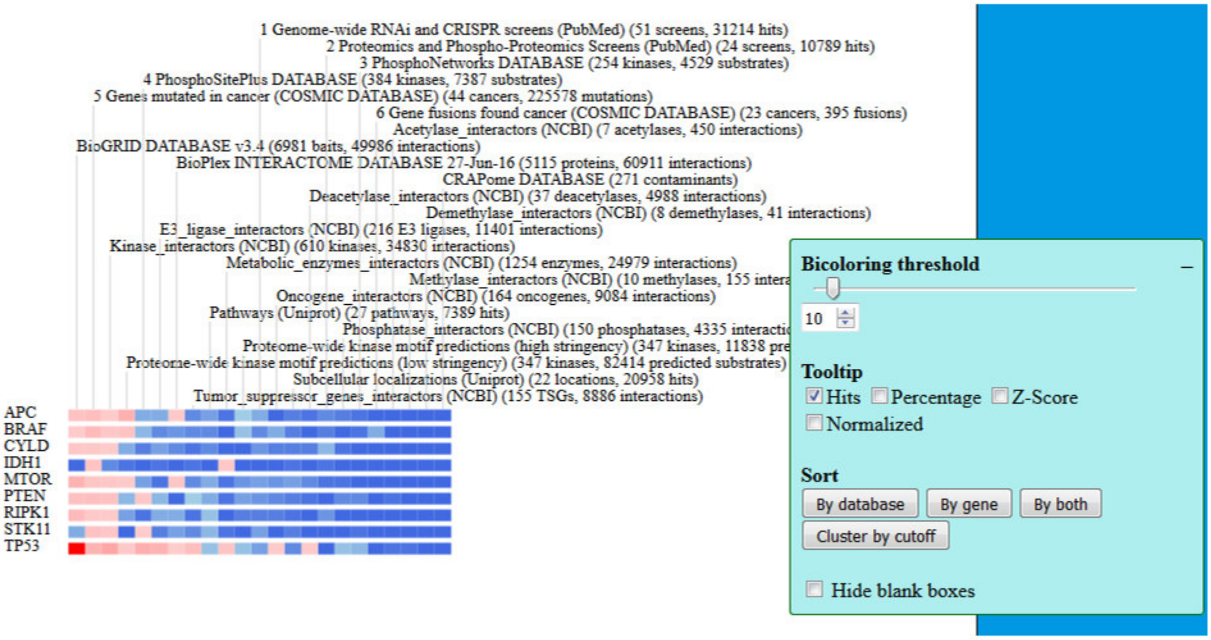

Supplementary Figure 10

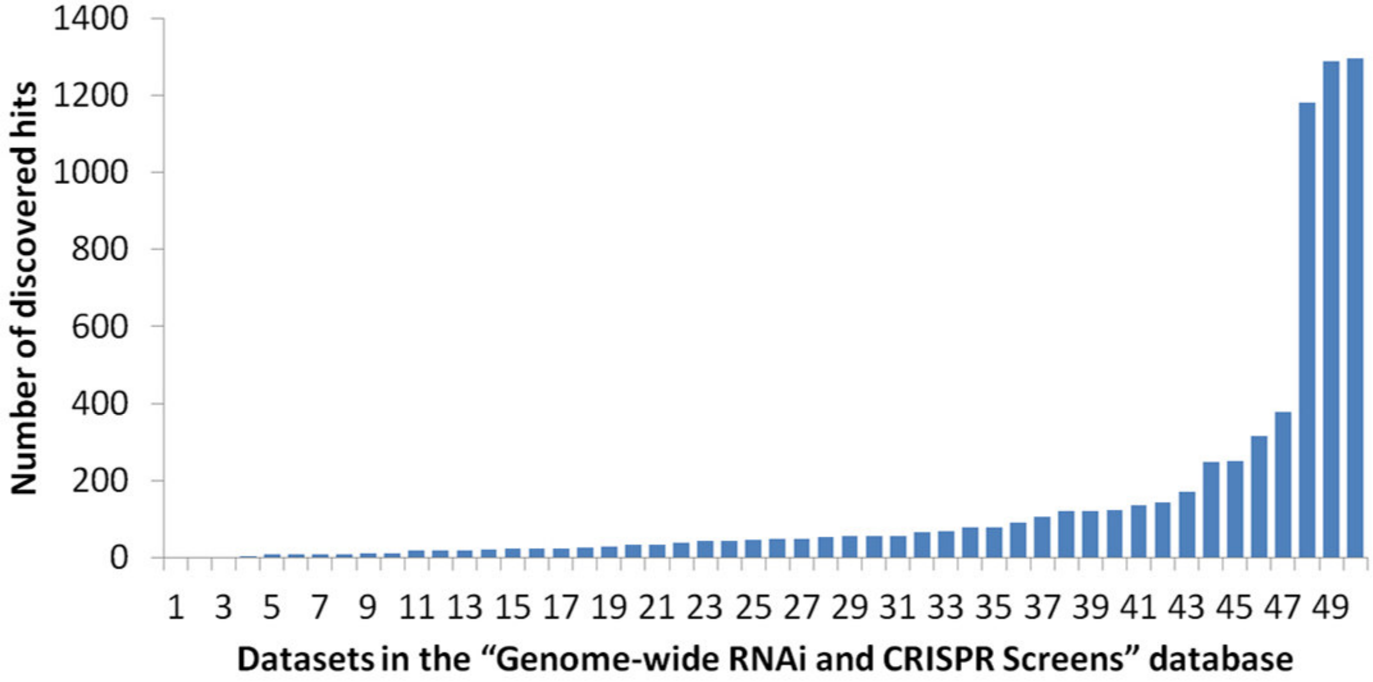

Supplementary Figure Legends

Supplementary Figure 1. CrossCheck user interface.

Supplementary Figure 2. Schematic of CrossCheck architecture and data flow.

Supplementary Figure 3. CrossCheck processing times. Indicated numbers of random unique gene symbols were used as input and were processed either online (www.proteinguru.com) (A, B, E and F) or on a local Apache server (C and D) using reference databases of indicated sizes. Times to finish the server-side process and produce the output files were recorded. Processing times as a function of increasing database size were given for a query of 5355 (E) or 100 (F) unique gene symbols. 10 iterations were done for online and 100 iterations were done for local server tests.

Supplementary Figure 4. CrossCheck Overview function analysis for the indicated genes, with sorting by the highest number of hits found in the CrossCheck reference databases *and* by the highest number of hits in a database for queried gene list at bicoloring threshold = 2.

Supplementary Figure 5. CrossCheck Overview function analysis for the indicated genes, with sorting by the highest number of hits found in the CrossCheck reference databases *and* by the highest number of hits in a database for queried gene list at bicoloring threshold = 10.

Supplementary Figure 6. CrossCheck Overview function analysis for the indicated genes, with sorting by the highest number of hits in a database for queried gene list at bicoloring threshold = 2.

Supplementary Figure 7. CrossCheck Overview function analysis for the indicated genes, with sorting by the highest number of hits in a database for queried gene list at bicoloring threshold = 10.

Supplementary Figure 8. CrossCheck Overview function analysis for the indicated genes, with sorting by the highest number of hits found in the CrossCheck reference databases at bicoloring threshold = 2.

Supplementary Figure 9. CrossCheck Overview function analysis for the indicated genes, with sorting by the highest number of hits found in the CrossCheck reference databases at biocloring threshold = 10.

Supplementary Figure 10. Illustration of the high-throughput capability of CrossCheck. Cross-referencing of 2306 essential genes from Wang *et al.* discovers 9411 common hits in 49 out of 50 databases.

Supplementary Table 2. Kinase motifs used in the proteome-wide kinase predictions (PKSP) database. Motifs were generated using phosphopeptide information provided by the PhosphoSitePlus website (www.phosphosite.org). High, medium and low stringency motifs consist of 15, 11 and 9 residue positions with the middle (8th) residue as the phosphorylation site (yellow highlight), respectively.

Kinase	Motif
AAK1	[LS][QR][IV][TV][DS][EQ][KV][T][GK][DQ][IL][GI][VW][DR][QR]
ABL	[AGKLPQRSV][DEGKLPTV][EGPQRST][AEGLPS][ADEGPSV][ADEGPSV][DEILPV][Y][ADEGNQSTV][AGNQRSTV][LPV][EKPRSVY][AEGKPSY][AEGKNPS][AGKLPRST]
ACK	[AEILQR][EFKLPSTY][ADPQRT][EGMNST][CDGKR][CDGLNRV][DHIKMY][SY][ADEGNVW][AFMPT][IKLMPQT][EILN][ADGHKL][DHKLQRTVY][AGKLNPR]
AKT1	[AEGLPQRS][FGPRST][R][GKPQRS][R][AKLRST][AGNRST][ST][AFLMSV][ADGPST][AEGLNS][AGLQRS][EGLPRS][AGKLPRS][EGLPQRS]
AKT2	[AGKQRT][AEGKLMPRT][KQR][AEKQRS][KR][LRSTV][AGKLNPRS][ST][AFGLPRST][AGNPRST][ADFGLQRST][EGIKLRS][ADEGPQRST][IPQRST][ALQRSV]
AKT3	[AGHPRS][AEGKPQ][KLRS][DEMPRS][AR][AKST][AST][ST][AEFRS][ALNPS][ENQRST][DEPSV][CDHIP][DIKQRY][LNPRV]
ALK	[ADFLQRS][EFGKPRV][DGKMPRSV][AGKQRS][ACEGQRV][ADGHKNPQR][EILPTV][Y][AEHLPRTV][ADFKNQ][AIPRSTV][CGKLTY][AFGLRTY][DILMNRSV][ACFKLQS]
ALK1	[AHST][QST][INS][EGHT][PS][CIST][GQS][ST][NST][HPQS][CMS][AIST][GPT][CS][ST]
ALK4	[PV][DK][LT][TY][NV][DE][EL][T][NT][DE][ES][T][T][DS][KS]
AMPKA1	[GPRST][AKLPST][ILMR][PRS][KLR][LRSTV][AGPQRS][ST][EFGLPSTV][AELNPST][ADEGNS][ILSV][AENPRSV][AEGLPQRS][AGLPQRS]
AMPKA2	[ADFGILNS][GHKLPQST][EFGLMT][DEHLPRSV][DKLRS][MPRST][AGLMPRST][ST][AFGILNQS][ACLPSV][AGHQST][FILPSTV][EFKLNSTV][AEGKNSV][FGKLPQSTY]
ARAF	[EGLPQV][EFIRS][DEGRS][GLQS][LMR][AHIRS][DLMNRS][S][ADFMPY][AEPTV][AFGNP][AGNSTV][DEFLRT][AESVW][ADFGKY]
ARG	[ADEGIKNRY][AEGILNQRST][ADGMNPSTV][ADEGLNPRST][AEGKPSV][ADGILPRSV][IKNPRTV][Y][ACDEGKLPQST][AKLNPQRST][DFHIPV][ADGHKLPSV][GILPSTVY][AEFGKMNST][CDGKLPT]
ASK1	[AGILQT][DGISTV][DGNST][EPRSTY][CFLM][GSTV][DEGKMS][STY][EFLPRV][ASTVY][GKLTV][RSTV][ALMQT][DLQRSY][APRVY]
ATM	[DELPST][DELPQST][DEGKLPQS][DELST][EGLPSTV][DELPQST][ADEGLS][ST][Q][ADEGPS][DELQST][DEILNPQST][DEGLQSV][DEKSV][EGLPQS]
ATR	[ADENPQS][AEFLQSTV][ADEGLPSV][ADEGKPS][AELPTV][AGPQST][ADGLPS][ST][Q][AEGPS][AEPST][ADGLPQS][ADEGIKLS][ADEKST][AGLQRSV]
AURA	[AKLPQRST][AGLPQRS][ANQRS][EKLST][GKRS][KR][AKLRST][ST][LRV][AGLPQSV][AGIPSV][AIKLNPS][ADGKLPRS][DEIKPRST][ADEKPRST]
AURB	[AKPRST][EGKPRST][EKQRS][AGKLRST][AGKRS][KR][KLRSV][ST][AFGLPSTV][AGLPSV][AEGLRSV][AKPRSTV][AEGKLPRST][DGPRSV][AELPRST]
AURC	[ET][KL][QV][PT][AS][R][KR][S][KT][GL][GR][K][AP][KP][PR]
BCKDK	[DET][DRVY][DRS][IKST][AGIT][CHVY][HKRT][S][FTV][DISY][DEKN][DQTV][GNS][ARSY][AILW]
BCR	[LV][TV][LT][VW][AT][ES][DN][ST][AQ][DG][DE][EL][AC][DER][AS]

BCR-ABL1	[ADEFILTVY][DEGLPQST][ADEMNPQR][CDEFINPQRSY][ACDEGKPQ][EGHKPRSTV][ADEFGINTY][Y][ADKRSTVY][GKMNPQSTV][DFGKLPV][CEKMQRV][ADEFLMNPSTV][EFGHKPQRST][AEGHNSTV]
BLK	[GPQTY][ADEPT][DET][ADENQ][DEKT][GINPT][GILT][Y][ELMS][ELTV][LP][MNP][EHLP][NPQRV][ADQ]
BMPR1B	[GHS][NPSV][HPRS][CHINSV][NPRSV][CIPRS][CIMSV][S][MSV][MSV][S]
BRAF	[EGLTV][FHIPS][DGQRSV][DQSV][LMPRS][AGIS][DKNY][ST][AFIMPT][AEPTV][GNPST][AGPSTV][FHRST][LSVY][DGISY]
BRK	[AEPRTV][ADEFGLV][LPSTY][AERS][DEMQT][DEGHTV][DLPTV][Y][DEGLQSV][EKRVWY][EHPV][DLPQT][DEGL][FLNQ][GQSW]
BRSK1	[MR][AE][AN][DH][GT][S][DI][S][LS][EL][FG][EF][RV][LT][C]
BRSK1ISO2	[AKLNRSV][DEGKLRV][ADEGLMRV][DELNSTY][FGKLRS][CILS][DEGKPRV][ST][LMPST][CEHPT][EGHINY][ADFLNSY][CEKLPV][HILNP][CEQRY]
BRSK2	[KS][DK][GI][EK][FR][LQ][LR][ST][MS][CT][GL][RS][GP][GN][RY]
BTK	[DEGLRS][AEIKLRSY][DHIKLQRSVY][AEIKLQSVY][DEIKLNQRSV][ADFQRSV][DEGLMRS][SY][DLPSTVY][DEKLPSVY][CEGHMPS][EHLPQRSV][AEGHIKMNPQR][EIKLNQSY][ADEGKMNQRSV]
BUB1	[AFKNQRS][AHKLMQSV][AEIKLPQV][FKLNQRST][CGKPQRTV][AKLRTV][AIKLPRS][ST][EFGIKPQSV][AFGHKLPS][AEGHKQST][ACGLNS][EGKPRSV][AEGKNVY][KNST]
BVR	[ESW][DQS][EGS][SV][EHT][ETV][ADK][TY][FI][CR][GQS][EFT][LPS][DNS][ASY]
CAMK1A	[EFKLNQRW][AFGKPQR][FKLPTVY][AKQRSTV][AKR][AKLNQRT][AGLPQRTV][ST][ADEFLQST][AEPRS][AEFGLNRS][AFGHLPQV][AKLMPQT][ADFLNQSVY][ADFLNPQRS]
CAMK1B	[FNQR][AIT][FLR][GMRT][DLRV][GNV][DGS][ST][EGMS][DGIK][DGLY][LMP][ALT][ADKN][AIKN]
CAMK2A	[GKLPRS][AGKLRSTV][AGILRS][AEGLRST][KRS][ALQRS][AEGKLQSV][ST][EILSV][DEILRS][ADEGKLRS][ADELPS][AEGKLS][ADGLNPRS][ADEKLPRST]
CAMK2B	[AGKMRST][GLPST][AGKLMRST][AELPST][AKPRST][AEGLNQST][AGKLNQRST][ST][FGILMSTV][ADEGINT][AEGLPST][AELQST][GKPTV][AEGKRST][ADEFGIQT]
CAMK2D	[ILRSY][AGHNS][GKLQ][HKSTV][KRS][GNQRT][EGKLSV][ST][FGILRSV][DEGT][ACES][AEGKLMN][GHKPQT][GPQV][AERVY]
CAMK2G	[CKLRT][DGNQ][DFLNR][LPSY][ALRT][AEPV][AEKM][S][AELMP][EGLM][EHNTY][AFKMR][ADKQT][AEQS][ADMTV]
CAMK4	[DEPRS][EGIPQRS][GL][AELQSV][KRS][KNQRST][AGHPQS][ST][EFGLMSY][APQRSV][EGKLNST][FILSV][DKLNPQT][ADELNPSV][ADLMQSV]
CAMKK1	[DEGLSTV][DEGHKMPRV][DGKPQRS][CDEQSTV][ACFLSV][ACLMSY][AEGIKRS][ST][ACEPSV][ACGSW][GHSY][CDLSTV][ADEPTV][AEGHNPT][AESY]
CAMKK2	[DGMQSV][CDELQRT][EGKPSV][ENPQSV][AFKPST][ACLPVY][ACDPRS][ST][CES][ACGPVW][CGQST][DPQS][ADPS][CENQRTV][DEPSTY]
CASK	[KLPV][HKLQV][ACEKP][EKSV][KLQS][AELPQ][AKNPS][S][AKS][AEKNP][KNSV][KNS][AKLNS][GKNP][GKNV]
CDC7	[IKLRS][RTV][DEKNPS][EFKQST][ADELPS][ADEGLST][ADEHS][S][DEFPQS][EGMRST][DEGRS][ADGKLST][ADEGLMST][AELMNST][AEGLPRS]
CDK1	[AEGKLPSV][AEGKPST][AEGLPS][EGLPQST][ALPSTV][AGLPSTV][AGKLPRSTV][ST][P][AGKPRSV][GKPRS][AKLPRS][AGKLPRS][AKLPRST][GKLPRST]
CDK10	[LP][DK][GS][GM][CL][LP][AD][S][MT][CP][PS][AV][LS][ST][PS]
CDK11A	[STW][DET][DES][ES][EVW][DTV][DS][S][CES][CSV][DGS][GQS][PQS][CIP][HIS]

CDK12	[ST][P][QS][SY][PS][PS][TY][S][P][ST][SY][PS][PS][TY][S]
CDK13	[ST][P][QS][SY][PS][PS][TY][S][P][ST][SY][PS][PS][TY][S]
CDK14	[GKPTY][DERV][DKLNP][DEKPS][DIRT][ADENQ][ACDFL][ST][DGRT][CEMNT][ADENQ][AFLT][GKQT][CEHQ][AGIMN]
CDK16	[DFN][LPSV][FRT][EILV][DKPS][EINR][CGIW][S][P][ADPS][IKLP][EIMP][INRS][GMP][DFV]
CDK19	[GPT][AP][A][ARV][PV][AIP][GSV][ST][ELS][DL][EGP][ADT][ADV][AD][ADK]
CDK2	[AGLPRST][AGLPST][AGLPST][AGLPST][AGLPSTV][ALPST][AGLPS][ST][P][AGKLPRSTV][KPRST][AKPRS][AGKLPRST][AGKLPRS][AEGKLPRS]
CDK20	[GI][RV][PS][KV][PR][PT][Y][T][DH][EY][V][SV][T][LR][W]
CDK3	[FGIKLPRSV][CFGHILNPSY][GILNPRST][ADELNPST][GIKLNPRTV][LMNPQRSY][AGIKLPSTV][ST][PY][DEFLNRSY][HKLMPV][AEGIKLQS][ADLMNPRS][AEFHKLSY][DGIKLPQ]
CDK4	[ELPST][GIPSV][AGS][GNPS][GILPRS][KLPSY][LPRS][ST][P][ALPRSY][KPRS][GIPRSV][KNPRSV][AEKLPRS][GKLPRST]
CDK5	[AELPST][AEGKLPRSTV][AGKPST][ADELNPST][ALPRST][KLPSTV][AGKLPRS][ST][P][AGKLPQRSV][HKPRS][APRS][AGKPQRS][AGKLPS][AKLPRST]
CDK6	[EILNPST][DGIKPTY][EGILPRSY][GPSV][IPST][LPSY][AGIKLPRS][ST][P][AILPRSTY][KPRS][IKPRS][GIPRSTV][GLRS][GKPRTV]
CDK7	[EGLRST][EIKPRSY][DEPSTV][EFPSV][AGLMPRS][ALPSTVY][AELPSTY][ST][DEPS][AEGPSTV][PQRSVY][LPQRSV][AEGPST][ELPRST][EGPRSW]
CDK8	[DNPSTY][ADEKLNQSTV][DGHILNQS][DELMNPST][ADFILNPY][HNPRY][EGHKLMNV][ST][KPST][AEFGKLNPQRT][AEGHKPQSTV][AFGKLMNRS][ADHLNPSY][ADEFLPSW][ADEGLMNRST]
CDK9	[GLMNPST][LPQSTY][GHIPQS][NPRSTY][GNPTY][AMPS][GLMPQRSY][ST][NP][ALMNPQRST][HIKPQSY][GNPRS][GPQS][AEGLPQSY][DGKPRST]
CDKL5	[ENP][AGLN][ALNP][INV][ARY][MNPT][ENRY][STY][EKPV][ADSY][NQTV][ARTV][QRTW][EKRY][GRTW]
CHAK1	[DEGKNPS][DKLNST][GKLNQRST][AEIKLPQRS][AEFKLNST][DEFGKMRSV][DLNPSTV][ST][EIKNSV][AEFKNPRSY][AEFGKLRS][AEIKLPS][EIKLPQRST][AEIKSV][DGKLPTV]
CHK1	[AEGKLPRS][EFGKLPRS][IKLRST][AEGKRS][KR][ALNPRSTV][KLPR][ST][AEFGLQS][AEGLQS][DEGKLPST][ADKLSV][ADEKLPS][DFKLPRSV][EKLNPQRS]
CHK2	[EGKPS][GKLPS][GKLPTV][AKLQRS][AGPRS][APQRSTV][GHKLPRS][ST][AFGKLPQT][EGKPRSV][ADGHKSV][AGKLPTV][AGIKLRSV][DLPS][EFGLPST]
CK1A	[ADEGKS][ADELS][ADGLMNQS][DEGQST][DEGST][ADES][DEGLS][ST][ADEGLQSV][ADEGLS][DEGS][AEGKLS][DEGLPS][DEGLNPS][ADEGLPS]
CK1D	[ADEGPS][DEGSV][AEGKPSV][ADEGLS][DEGRS][AEGQST][ADEGLS][ST][DEGLPQSV][ADEGS][ADEGLS][ADEGKLPS][EGLPRS][EGLS][DEGLPRS]
CK1E	[AEGS][ILQRS][CGPSTV][AEPST][EGPST][AGPSV][AEGPST][ST][ALMPQRV][AEPSTV][DGLPRST][AGKLPSV][ADEGLPRST][LPRS][DEGPQRS]
CK1G1	[DFPS][DGS][CDFGS][EFLS][CDST][ACFKS][GLS][ST][AILSY][ADFLS][ADEPS][ALMP][DFILS][EFILS][DLNPQS]
CK1G2	[EKS][IKS][EGL][DKR][IRT][EGH][DG][ST][GM][GV][S][GLY][GSV][IS][GKY]
CK2A1	[ADEGKLPRS][ADEGKS][ADEGLRS][DEGLS][ADEGS][ADES][DEGLS][ST][DES][DEGS][DES][DEGS][DES][ADES][DEGPS]
CK2A2	[ADEGQV][ADEGSW][DEPV][EHT][AEIL][AEGLP][DEGQSV][ST][DEGLQST][DES][DE][DEGLT][DEGHMTV][DEQRST][DEGHILS]

CK2B	[KMV][AS][EGP][LMY][HST][DSW][DLN][S][D][AE][DS][LST][GLS][EK][AG]
CKB	[GPT][CHY][ILP][GLS][NTY][CIL][GLP][ST][CGN][LP][GRS][ANT][GL][GLV][HRT]
CKM	[EGT][CHY][ILP][GLS][NTY][CIL][GLP][ST][CGN][LP][GRS][GNT][GL][GLV][HRT]
CLK1	[DEIKMQR][ADEGKNST][DEKLQRT][ADEKPRSWY][DKRST][ADGKPS][AGKLPQRSV][ST][DKLPQSTV][DEFKPSTV][ADGIKLNRS][ADHIKPRT][AIKLPQS][AIKNPQRTV][EILPRSV]
CLK2	[DMR][DEKN][KR][EKRY][DKR][DKP][PSV][S][MPSV][DFRV][DIL][HIK][KRS][GKRV][AILV]
COT	[DILV][GISV][DGKLQR][DEQSY][IKLMR][AIKRSVY][DFKNR][ST][FLMPRV][AEPRV][EGKNQR][DIST][FLPR][DKLNQSV][AGSY]
CRIK	[K][KR][PR][PQ][QRS][ARS][AT][ST][NS][NV][FV][AF][AM][FM][DF]
CSFR	[DEKLNPS][EILPSY][EHKLMQRY][EGLNQR][DEGKPV][DKNPQSV][DKNST][Y][AIKQTV][EFNRV][CIKLMR][DGHPRW][KLNPS][AEGISTV][FGIKQRS]
CSK	[FMPQTY][QRSTV][ANST][DMT][DEFS][EGPTV][AEQV][Y][QST][EFIPQ][GILQV][DEHPRY][DKNQV][LSTV][DIS]
CTK	[EQY][DPT][ANT][NT][DEH][GIT][EQT][Y][AQ][DQS][ILQ][NPQ][LT][PS][KP]
DAPK1	[AHKP][KRS][KLPR][EKLQRS][RSW][AKQRS][FPQT][ST][DFNSV][DELRV][FLTV][AFIL][LMSV][FKL][ACDIL]
DAPK3	[EKPQRT][DGKLPRY][GPR][EKPQR][GKQRS][AGRS][AKMST][ST][DINRST][AEGKLNTV][FQSV][ADEFKS][AFLMNRS][EFMSTY][DEFHTV]
DLK	[IKL][KQRSV][DGPRT][APQS][ILQR][AKRV][ADKST][ST][GINRS][ADGNV][AFKV][AFGT][ACMPR][DFMR][ADFKP]
DNAPK	[AEGLQS][AELMQST][AEGKPST][AEGLQST][GLNPQSTY][AGPQSV][ADEGLPSY][ST][LPQ][ADEPQS][ADGLPST][DGKLQST][DEGLPQSV][DGLPST][EGLPQST]
DRAK2	[EPRS][EFKNR][DEIRS][CDFGKPS][CDERST][CDLNS][GKPRV][S][CGKLPSV][CGHLRSV][AEFGLNS][GKLRT][EFLRSTV][CDFPT][DGKPRT]
DYRK1A	[AGKLPS][AEGLPRST][GIKPRSVY][AGKLPRS][GLPRSV][APRSVY][ADEGKLPRSTY][ST][P][AGIPQRSTV][DHLPSTV][ALPRSTV][EGLPRST][DEGIKPRST][GKLPRSTV]
DYRK1B	[AEIKLNPSVY][ADEGHMQRTV][AIKLPRTY][ADEGKPRSVY][ACFKLRSV][ANPQRSTY][ACGHIKLPS][ST][DKPQSV][AELPRSTV][DGLPQR][ADEGLRSV][DEGILPRV][DFGIKLMQS][DEHILMRSV]
DYRK2	[AGLPQT][AGLMPQRS][DGMNPRSV][DENQRS][AGLPR][AKPRS][AGLNPRTY][ST][PV][AGLQRS][DGKLPR][HKLPRST][AELNPQRT][AGILPQRS][AGNPQRT]
DYRK3	[EHLP][HILPY][LQRS][EKPSY][LR][LPRY][GNPT][STY][IPSVY][ADLQR][FHKLS][IQR][EFKLS][EILNY][DKR]
EEF2K	[AEGKRST][AEFGKLPSY][ADEGHLQT][EILNQRSV][ADFGLPRST][AEGPRTY][ADEFGKLPS][ST][ADEGILPR][EFGKLSTVY][ADKPRST][ACDEGKLPS][DEGPQT][EKLPRSV][DEHKLPQRV]
EGFR	[ADEGKST][ADEGLPS][ADELT][DEGKNS][DEGLQ][ADEGNPQSV][DEINPS][Y][ELQV][DENPQRS][AFLMPV][EFNPT][EGKLPQT][GKLMNS][AFGKPS]
EPHA2	[AEGKPQRT][DGHLMQSY][ADEIKLMQSV][ADFIKP][AEGILMNPR][ADEHIKMQST][AENQTV][Y][EFKMSTV][DEGHLST][HKLMNPRS][DFGHLNS][AEGKLMQT][AKLQTY][ADEILSV]
EPHA3	[EKT][DLY][DPV][DGP][ELP][AHR][AT][Y][ETV][DT][PR][GHT][GQT][AKY][EIV]
EPHA4	[EILQT][ADENY][DIQV][DGIKP][EGMPV][ADFLR][ALRT][Y][EKQTV][DET][NPRY][FGNR][DGQT][AKY][AEISV]
EPHA8	[FL][AS][AY][EG][EP][HR][PT][Y][EW][EN][MP][GT][NR][AQ][DG]

EPHB1	[DPT][DGKP][DGKN][DQSY][ACER][AENV][FGNP][Y][ADFL][KLP][ST][KS][FL][GIN][CEV]
EPHB2	[DIMR][KPTY][IKPT][DGIS][EMPS][ADFGK][AIST][Y][DEIQT][DKL][AGIP][CEFNP][ERST][AKVY][DEPV]
EPHB6	[DRSTY][DGKRTY][ADGILR][DGLRS][EGLNPV][KPQSTV][KPQTY][Y][EITY][DGIS][ADPRT][LNPRS][EGQST][ACLQTY][EGIKY]
ERK1	[ADEKPST][ADGPST][ALPRSTV][ADEGLPS][AGLPRS][LPSTV][AGLPRST][ST][P][GLPRST][ALPSV][AGLPST][ADGLPST][AGLPRST][AEGLPRST]
ERK2	[AEGKPST][AEGPST][AGLPST][EGLPST][AGLPS][LPSV][AGLPRST][ST][P][AGLPRSTV][ALPS][AGPST][AGLPRST][AGLPRST][AEGLPRST]
ERK3	[EPY][MPS][EHY][KNR][AGR][AHL][LNV][S][ELY][DFG][ELS][PV][PTV][KSV][EVW]
ERK5	[ADGLPQS][AGNPS][ADLPST][EGPSV][ADFGPS][AFGLPSV][AFLPS][ST][AP][AEGS][ADLPS][DKLPS][AEGPS][ADLS][GLS]
ERK7	[ADEGKRV][ACDGHKNP][DEGLQS][ADGLPV][EKLPQSV][AGLQST][AEGLPRSTV][STY][EHPRSV][ADEPRSTVY][ELPQTVY][AEGKRTVY][GHLPQRTW][GLPRSY][EHIKPRVW]
ETK	[DKRT][DIKMV][EGIKSV][ADEHLNS][ADKQST][DEHNST][DHITY][Y][EKNQSY][KLNSV][LPSTV][EGNST][AEGKN][GLPQ][AGLPQV]
FAK	[DEFQRSY][DGLPSVY][AGNPST][DES][DEMST][DEPS][DITV][Y][ADESV][EFNRSTVY][AILPTV][AEIPQS][ADEGKNPS][DEKLNPQS][DEGKLS]
FAM20C	[CIP][LNP][EIR][NRV][AH][EPT][DLR][ST][AG][DE][DFN][DIS][PRS][EK][DFR]
FER	[ADPRT][AEPQV][AEGLNPV][DEMNST][ADEGKL][ADGIKPQSV][ENPV][Y][DELQST][DEGKST][DGSTV][AEGKRT][AEFKLRSV][IKLSV][ACEIKLQS]
FES	[DEPQRSTV][CENPR][ADEGILS][ADEFMTV][DEQSV][GNPST][DFILRV][Y][AEQST][ADEFR][ADILSTV][EGKLQRY][AEGKLMS][EIKLNPSV][DELPQR]
FGFR1	[ADKLR][DILQR][CGHILP][CDGHLPSTV][DGHILNSV][CDILS][DEILY][Y][CEKMNQY][EKLPV][AKLNPSTV][EPRST][DEGNPST][EGHNPS][EGLNRW]
FGFR2	[AIRT][GLRV][ETV][ADNT][EKLN][EFIP][DEMN][Y][FGLQ][ADE][DFL][APSY][EQ][GLP][ALR]
FGFR3	[ADRT][DGINRV][CEHPTVY][DHKNT][DGHKLNT][DFGILTV][DEGKLMNY][Y][FIKLMQVY][DEHIKY][ADFKMTV][AEPQRTV][EGLNQT][CEGILNPV][AGLRSVW]
FGR	[CDFMRSTVY][DEKLRTVY][AGIKMPSV][EGNPRTV][DEIKQST][DEIKPQST][ADELNQTV][Y][ADEGKNQY][DEHKNPY][AGILPQSTV][DIKPQST][DGHKPQS][DEGILPRTY][AEKNSV]
FLT3	[ADKPSTVY][DEGIKLRST][ADEFHMRS][CELNRS][CDEFLP][ADHNQSTY][EFLMNPQT][Y][DFIPQTVY][DILNSTVY][FKNRTV][DGLQRSW][DEFGNPQS][AEFHLQRY][EGLPRSVY]
FRK	[GHIKN][DGKSV][DLT][HKNPT][AET][ADNTV][IRST][TY][EFGLP][ENPS][IKPRS][EHNQ][DEFG][AIKLN][AEFKV]
FYN	[ADEKPRST][DEKLQS][DENQST][DEGKPQSTV][ADEGQ][ADEGLNPS][EGILPTV][Y][DEGQSTV][ADELNQSTV][DILPSV][ADEKNPRS][AEGKPS][EGKLNPQSV][ADEGKLPQS]
GCN2	[ELMN][EGIR][AELR][GLT][ALMS][EFPT][GLV][S][DEKR][EFRW][EFRT][GIP][AGNR][AMSY][AIQV]
GRK1	[ADPT][DELQSV][AGIKSV][DGKRS][ADRST][ADEFRST][AEGLPT][ST][AKTV][IKTV][AEGKRSV][AGNSTV][AKSV][AQTV][EFGKNPQSV]
GRK2	[AEKQRS][AEGKNSV][DEGNRST][DEGLNRS][DEKNRST][DEPSY][DEKPSV][ST][DELSTV][ADEGNRSV][DENQST][DGSTV][DEGQRST][DEGKQSV][ADEGLNQRS]

GRK3	[ADEKQRSV][ALST][GPRST][EGR][EGNRST][EQRST][ADERST][ST][ERSTV][AEGRSTV][DEHNT][PQRT][DGRST][GLRST][AGLQS]
GRK4	[EGKLMRSV][DEFILMPQTV][ACDGIKLNSY][DEILQRTY][ADEFKLMTV][ADELNQRSVY][DEFIKLNPQTV][ST][DIKLMRV][ACDEFGKLNRSV][AKLPRSTVY][AEFGIKLMQSV][ACEIKLQRSY][EHKLPQRTY][ACGIKLQSTV]
GRK5	[ADKLV][ADLNY][DGIQY][DEGIN][EGNQT][FGNQY][DFGPSY][ST][ENSTV][DGKNV][GKN][DGNSTV][GNTV][DEGLN][DEGKLQRTY]
GRK6	[AGKLPTV][ADKLPSTY][DGIKLRSVY][AEGILNPSVY][DEGKLNQRS][EFGHIQRSY][AEFGHLSY][ST][EGKLNSTV][DEGHKLNSV][EGIKNTY][AEGILMNRSTV][AEGLSTV][AEGKLNS][AEGHKLQST]
GSK3A	[EGKPRSTV][AKPSTV][AEKPRSV][AEGST][GKLPSV][ADLPSTVY][AGKPRST][ST][PR][AEGPRSV][GHKPSTV][ALPST][GKPS][ADENPST][AGPRSTV]
GSK3B	[AGKLPS][EGLPSTV][EGLPRST][EPST][EGLPRSV][ALPST][AGLPSV][ST][PR][APS][AGLPST][EST][AEGLPRST][ADPST][AEGPRS]
GSK3B ISO2	[RS][GL][ES][P][NS][PV][PS][SY][IP][CI][ES][KR][TY][PY][LR]
GTF2F1	[EGRS][EGM][ENSV][MPS][RSV][GPS][GNPS][ST][PR][PS][AGP][EPT][GP][GPRS][APSV]
GUCY2D	[KRSV][RSV][KQSV][GLQV][AGSV][QRST][GRS][S][ALRS][ALS][ADST][ILRT][ARS][AST][ARSV]
HCK	[ALRSTV][AIKLNPRV][AEIKLMN][AEKST][DGHKPV][ADLNPS][AEGLNSTV][Y][DHIPQTV][AFKPY][HIPRSV][AEPQV][DGINRS][AEIKRS][DHKLNQ]
HER2	[ADGPS][ACDPST][ADFISTV][DEFGLP][DENPQ][ALNPQ][ELTY][Y][ALVWY][ADGLNVW][ADFLPQ][DPQR][DEGNQTV][GPSV][ELPRV]
HGK	[ALQ][G][R][D][K][Y][K][T][L][R][Q][I][R][Q][G]
HIPK1	[PT][GS][QV][EQ][PR][EM][GP][ST][P][P][IL][AE][ES][RV][AK]
HIPK2	[ADEKPQS][EHKLPRSTV][EIKPRS][DEGPQRSV][AEHKPST][AGPSTV][AGPSTV][ST][P][AGKPQST][LPQRST][AEIKPQRST][EGPRSTVY][ADGIKLPRSTVY][AGPQRS]
HIPK3	[S][PY][LS][IS][HQ][MS][T][ST][P][LS][IS][HS][ST][RT][GT]
HPK1	[AIMSTW][HKQS][AFKMP][PQRT][ILPQRT][AGPQS][AMRS][ST][ALRS][AIPS][AGMPRS][IPRSTY][FILMNY][AST][AFILQW]
HRI	[GIMR][EIKN][GL][KLMT][GIRS][ELT][HLR][ST][EHRS][LRT][RSV][GIR][RTV][CGRS][ILT]
ICK	[ALMS][AKPR][GLPS][AEGP][RY][PT][DGLR][TY][STV][GKPS][EGPT][AKRS][AGLW][ARSY][PQR]
IGF1R	[DEFGRSY][ADEFGILPR][DEIKMPSY][CDEGNSTVY][EGNPRST][DGLPST][DEFINPY][Y][AEFMRY][DKNRST][ADEGKLMV][ADEGNQSY][DGKLMNPVY][GKLRSV][GKLPRSY]
IKKA	[DEGLPRS][AEGHKLPV][DPSV][DEGNPST][DGLPRV][AFHLRST][DHLPSTV][ST][GILPV][AEGIKLS][DEGLPS][EIKLMPST][ADEGPQSV][AEFGKLNPS][EGKPRSTV]
IKKB	[ADLNST][GHLPRSV][DGLPS][DEPST][ADGLS][FHLQRS][DLS][S][GILP][AEIKLPQS][DEGLS][AELMS][ADEKLMPQS][AEFGPY][ELPQST]
IKKE	[DGILRSV][DGLQSV][DPRV][DEKLS][DEGPRS][FHLMQS][DPSTV][ST][FILMTV][ACEKLPS][DELNSY][AEPST][DEGLQS][EFGKSTY][DEKLSV]
ILK	[ADKPQRV][EHKLPR][AHLPQR][EFHPQSY][AEGPQRV][AEKMQR][ADFKPSTV][ST][GINQSTVY][GKNPQSV][AFRVY][ADFRSTV][AGLMRST][ADFKMQRST][ADEFGQST]
INSR	[ADEFGNRSTV][DEGIKLPRS][DEIKMNS][ADEKQSTY][DEGPR][DEGNPSTV][DEGINQS][Y][DEILMSTV][DEKNPST][DGILMP][DEGNSTVY][FGKLPRSY][DEGKPRSVY

	][AFGKLS]
IRAK1	[GNQV][ACQST][AGIPQT][ADEIKQSV][AGHKLV][AFPRS][AGIMNV][ST][AILP][AKRSTV][AFQRTVY][AEKLPQST][DLRSV][AEKSTV][EILSV]
IRAK4	[FGKNPQY][AGKLNQST][AEMPQRSTY][AKNPQSTV][DGIKNPSTV][ADFNPQRST][DGILNPQST][ST][AKLMPQRSTVY][ADEKLPRSVY][AEILMPQRSTVY][AEIKPQRSTVY][KLPQRSTV][EIKLQRSTV][AEIKLNPQRTV]
ITK	[EFIKLQRS][DEGHKLMPRST][AEKLNQRSTV][EILNRSTV][EGHIKLNPV][AEGLNQS][DEFGHILMQV][Y][ADILNQTV][DEINSTWY][AEFKLMNQRV][EINPRSTV][FGKMNPQ][DHKMNPSTV][ADGILPVY]
JAK1	[DINPV][GHLNPRS][EKLNP][KLPQRS][DEGKQSV][EGKST][ADGPRS][Y][GIPSV][AGKNPQT][ALMPT][DELNQS][DGHILST][CEFIPR][ELMPSY]
JAK2	[DGILPTV][AEGLPR][EGKLPRY][AEFKLPS][AGIKLPV][DEKPTV][DEGR][Y][EIKLV][AKPQ][LPTV][DKLQSV][DIKPRY][AEIKNSY][ELPQRST]
JAK3	[ALSV][AGILS][GLPST][ADHIKLT][DGKPSV][DEKLMS][DEGY][Y][AHILSVY][KLSTV][LPRSTV][AEGKNQR][DFHLPR][ILPQR][GQRST]
JNK1	[AGPS][AEGLPSTV][ADGILPS][ADELPST][AGLPST][ALPST][AGLPQRST][ST][P][AEGLPSTV][AEPST][EFGPST][AEGLPRSV][FGLPST][AGLNPST]
JNK2	[GILPST][GKLPSTV][GILPRSV][ADLPS][AEGLNPSTV][AILPS][AGLPS][ST][P][EGLPRST][AELPRST][EFGKPRST][AEGPRS][AGLPRS][LNPST]
JNK2ISO2	[HN][MT][AI][DE][LV][NP][AM][S][P][LR][KT][HL][DF][SV][LT]
JNK3	[ACEGKLSTV][ADELNPRTV][ADEFGLPSVY][ADEPRST][AEFKLPT][AILPV][ACLPQSTV][ST][P][ADEGLPSTV][AEKLNPS][EFGKPRSTV][AEGHLMPSV][ADELPSTV][AEGHKLRSTV]
KHS1	[S][G][R][P][R][T][T][S][F][A][E][S][C][K][P]
KHS2	[AL][GT][DI][A][K][RT][KN][ST][F][CI][G][T][P][DY][WY]
KIS	[DGNPS][APRSV][DEHNPQRV][DEKPSV][EFGQRST][DENPST][GLPR][S][DP][EGMPQST][AEHKLP][EIKLRS][DKLPRY][DEILMNS][DGLQSY]
KIT	[CDEIKRSV][DEHIMNSY][ADGIKLPS][EILNQST][ADEGKNT][AEHMNPSY][AEIKLMNV][Y][EIKLMSVY][DNQVY][IKLMPQW][ADGIKLPTW][AHKMNPQR][ACFGLMSTVY][EKPQRSV]
KSR2	[DGLV][DIS][ADG][FQSV][ELMN][AGIK][DINT][S][EFMS][ALV][EGN][AST][FGNR][LNSV][EGY]
LATS1	[AEHIKLPQSTV][DGLMQRSVY][HLMPR][CDLQRSV][KLRV][ADGKLQS][ADEHLNPRSTW][ST][ACDEFLQRST][DEGLPRSTV][ADEGILNS][EGILMPQRSTV][DEHKLPQSTW][ADELMNQSV][ADGLMNSVY]
LATS2	[AGHLPQV][GLMQRVY][GHKL][ARVY][R][AIKQRST][HLNPRST][ST][ACEGMRSW][EGLPRST][ADEKLSV][FGILMPRS][FKLQSWY][HLMQSV][ACDILSY]
LCK	[ADELPRS][AEKLST][AEGLP][ADEPT][DELN][ADEGNPS][DEILQV][Y][ADESTV][AEGPST][AFILPRV][EPQRST][DEGKNPRS][ADEKLPQR][EGKPR]
LIMK1	[M][A][S][G][V][AQ][V][AS][D][EG]
LIMK2	[M][A][S][G][V][AQ][V][AS][D][EG]
LKB1	[KQSTV][DEGKLPSW][ADGPR][EKNQRST][FIKLPR][LRS][ADEGLQRSTY][ST][DFIQSW][CDKNS][DGIP][STY][KLPR][EHLNPS][EGY]
LMR2	[ENS][EP][GKL][EGR][KR][PQR][IL][ST][LPV][PRV][GPR][DLN][AS][AEG][KLQ]
LOK	[ALQY][DG][GR][DE][KR][KY][K][T][L][CR][GQ][IT][PR][NQ][GY]
LRRK1	[PS][LT][AE][EH][AI][GY][GK][ST][EN][I][EQ][LR][KV][KV][E]

LRRK2	[AGKNPR][GIKLNRW][AEKR][AFGKLM][AGIKLS][EGHIKRY][AGKLSY][ST][EHKLPS][GIKLRS][AHLR][DEKLQR][AGKLR][GLPQRV][ADEGILP]
LYN	[IKPRST][ADEGKLPTV][ELPRSV][ADEGKPST][DEKPST][ADEGNPST][DEILPSTV][Y][DEGLS][AEKNSTVY][ILPSV][DEPRS][DEGKP][KLNPS][ADEGKLP]
MAPKAPK2	[GKPRSV][AFGLPRS][FIL][GKLRSY][R][AQRST][FLPQRTV][S][EFLV][EGRST][ADEGLPSV][ADEILPS][AGKLPRS][ELPQRS][ADGKPR]
MAPKAPK3	[IP][FP][LP][AF][R][HM][AM][S][TY][ES][S][AT][NT][S][DF]
MAPKAPK5	[DFGIKLNPRTV][AEFGHINQTV][FHIKLMNTV][ADEFGLNPQRS][DFHLPRST][ACGHLPRSTV][ACHILPQRV][S][AEKLPQRVY][AEGIKLQS][ADELMNPS][ADEGILPQST][ADEHIKNTY][AKLPQRSV][ADEFGKLMV]
MARK1	[GKNRTV][ADHKRSV][EIKLQRTV][ADILPS][GKMQV][ACGIVY][DGKLRT][ST][KLNPTV][CDEGPQV][GIKLNV][AIKLSV][AGHKLPTY][DGHKLS][AKNPQSTV]
MARK2	[EFKNPRSV][AGLPRSV][DKLQRSV][GNPQRS][GHKRS][ACGIRST][AGHLRSV][S][CDELMPQT][DELPS][ADLNPS][DGILV][HIKLSTV][ADEGHLNRT][EKLNQRSTV]
MARK3	[LPS][AGM][KLM][INTY][DIRT][EGS][GNP][S][CLMT][P][ADEY][HLN][LST][MNP][LMNR]
MARK3ISO3	[QRST][GPQT][LRST][RST][NQRS][GLRT][FGRS][ST][KNRT][GLN][FLST][FST][KNS][KL][FLT]
MARK4	[PS][AS][LM][NS][GRT][GT][GNS][S][DLT][PST][ADN][L][HT][HNT][LST]
MEK1	[HR][DGLT][GHR][EFT][AGL][FIRTV][EL][STY][EIPV][AY][AGTV][ALR][ETW][ARVY][LRW]
MEK2	[HST][DPTV][GHLN][FNQT][GILS][FLT][ELS][STY][EPV][ALPY][PTV][ADLR][ETW][RSVY][DRSW]
MEK5	[AH][EQ][HY][FQ][MY][FT][EM][TY][EV][AY][TV][AR][TW][RY][RW]
MEKK1	[AEFILMNPQV][CGINQRSTV][ADGILPRS][ADEPQRS][GKLMRSV][AIKLPV][DGKMNRS][ST][FGIKMPR][AGLMRSV][AFGKNRST][CEGILMPSTW][ACDEFLNR][APQSVY][ADEGLSVY]
MEKK2	[ELQS][DMSY][DGNY][DGKT][GKL][FMR][GKL][ST][FIV][AEPT][GLRY][ALT][PRS][PRSY][ELTW]
MEKK3	[FIPS][DLMS][DGIK][GQSY][FLP][FLPV][ADGI][ST][IPV][AP][AKVY][GLST][MPW][DKP][AEQV]
MEKK6	[AGRT][DGHIS][DFNT][DEMPS][CDFM][EMT][EGMP][TY][FGPV][ATVY][GTV][ARTV][LTWY][QRY][RWY]
MELK	[DEGKNSTV][ADEGILNPQV][ADEKLNRSTV][DFKLMNPSTY][CDGHKNR][ACKLPQRSTV][AEGIKLNPQW][STY][AEFHLMPQV][ACELPSTV][CEGKLNPT][ADFGLST][ELPQSTV][ACDEKNPT][KLPRSTY]
MER	[FK][GIK][ILY][SY][GKS][DGK][DIY][Y][RSY][GQR][DGQ][GRY][IRY][AIR][AKQ]
MET	[ADEMRSY][ADEGILMRV][DEFHILMSY][DEKLNSVY][DEGKQRS][ADEKPSV][DEHNSTY][Y][CDELSTVY][DHIKNPSV][HLMPV][DGHKNPQST][AGKLNPQT][DGKLNPRST][DEGHKLSTVY]
MKK3	[ILMRT][DHSV][DGT][DESTY][DGLMV][AEITV][DGKMR][STY][GMV][ADTY][AGKTV][AGRT][CMPTW][DKRY][APRW]
MKK4	[AGT][GNSV][DFT][MS][FM][AEMT][DGMP][STY][PV][AHVY][ATV][RV][TY][RY][ARY]
MKK6	[AIKLRT][DGHQS][ADGLST][DEGPVY][CDGLMS][AEKTV][DGLMP][STY][GIMVW][AMVY][AKPSTV][AEPRTV][EIPRTW][DEIRVY][AINRSW]
MKK7	[AQST][CGNST][EFMRT][MNQS][FGMT][AELMT][AMPQ][STY][DLPV][AMTVY][DSTV][DRSVW][NSTY][DLRSY][DRSY]

MLK2	[AKNSY][AEFGPQS][AHKLNS][ALNPQST][FGLPRS][ADIPSV][AGLSY][ST][KPY][DIQRSV][AGHQTY][DKLPS][AGNPRSV][FGNQ][GLNSTVY]
MLK3	[ILQSTW][ADHKRSV][DGKPRY][EFLQSTY][EFILTVW][ADHLNQV][ADKMQR][ST][AFIMRSTV][ACDQR][AFGKLM][DGRST][ACMPRTY][ADGKNR][AEFGPWY]
MNK1	[ADEGMRTVY][AGLMPQSTY][AFGLNRSTY][DGKPRST][AGKRS][FGLNRS][CGILRS][ST][DLQRSTV][GKLRSTVY][FGKLNRSTY][GNRSY][FGNPQRST][FGNRSV][EGRSV]
MNK2	[ADEMRV][AGPST][AFGLRS][DGKST][AKRS][FNRS][GILRS][S][FLRT][DGLRTV][AGKRST][GNRST][GPRT][FGS][EGRSV]
MOS	[DGLPSVY][GILQSV][DEGQRV][ACGIQSV][AILMNRV][AEIMNQRS][ACDENPT][ST][FIMPQRS][AHLPQV][GHLNS][DHIKLSTV][ACDFGRS][ADGLPRSV][AEGLRTY]
MPSK1	[AEY][AEGQ][FQRS][CMRT][CEQT][AILRT][ILST][STY][LRVY][AEPQR][ADGLP][CEPVW][AEIL][AFLNR][FQSY]
MRCKA	[HKMQS][DELRS][DPRS][FGKQV][AKRTV][AGRV][FGQTY][ST][NPSVY][DTV][AFGSY][AINSV][CGMPS][FPTVY][DELPW]
MSK1	[AEGKRST][IKMRT][KLQR][AEKLST][AR][RS][KLPT][S][ADGPTY][EGPRS][AEGKT][EIKLST][AGLQRST][EGKNPRS][DGKLPRSV]
MSK2	[AEST][EIKMR][KLQ][ELST][ART][GRT][FGKP][KS][ADFTY][CGPR][EGKR][IKLT][AILQS][ELNP][ADPRY]
MST1	[ADGILNPRS][ADEGHNPSTW][ADEGKMPS][ADFGLPRS][ADGKRTW][DGKLNRS][ADGKLNP][ST][GILMQVW][EIKLPRW][ADGHIKMPRS][ADFINPQRTV][DEFIKLMNPR][AFGKLNPQRS][ADKLRSWY]
MST2	[DEFGKLPQ][EIKLNQST][EHKMNRSV][AHMNPRV][AEKLQRS][AEFLNRSY][ADEFGHKNPT][ST][FILMSV][DGIKLPT][AFGKLPRS][FGKLNST][ACGKLNPR][DEFKLNPQT][DEILPRSW]
MST3	[DRT][CHTW][LQV][FILQ][GIKL][KNRV][FNVY][ST][FKVY][DKV][GQRY][DFT][EPR][EFGK][ELVW]
MST4	[ELQRV][EGHLN][AELQR][DFLQRS][AGKQRV][AKNQRY][GKLPQV][ST][AFILV][AIKPR][AKQRY][ADINT][DFLPR][IKQRT][AEGKL]
MTOR	[DEGLNPS][GLPRST][GLPSTV][DEFLPRST][FLPRS][AGPSV][FGLPST][ST][LP][EGPSTV][AGPRS][LPST][GLPRSTV][AEGLPSTV][LPRS]
MUSK	[MNRY][AILNP][HIRY][ENPSY][AENS][ADIPV][DEIMY][Y][KQVY][AKRV][ADMR][DGIP][GLN][DELN][ADGIL]
MYO3A	[ISTV][NRST][AHLR][AHQ][KLR][EGRT][DNST][T][GNRS][EHMV][AGKR][RT][NPQR][FHT][ASVW]
MYT1	[EI][EK][IK][GI][EG][EG][GT][TY][GY][GV][V][VY][KY][GK][GR]
NDR1	[ADIKLNPQRT][GKLQRSTVWY][HRV][FKLQRSTV][ILR][ADGHINQST][ADEFHKLNRSY][ST][ACDEFLSTY][ADEGKPSTV][ADGIRSTV][AFGHILPRST][DELMPQSTW][ADGILQSVY][ADGILMRSVY]
NDR2	[KNPQT][GKQRY][DHR][KQSTV][FLR][ADLN][EHKQY][ST][FST][CDPSV][AGS][AGST][LMP][DLQS][GLMY]
NEK1	[DGLQRSTY][CEGKLPR][DELPRTVY][DEKLPQR][EFIPSV][AEGHPRTVY][DGLNPRS][STY][GIKLQR][EGLPRSVY][EGHKLPQRS][DGIKLMP][AEGHILTVWY][CEGHLNSTV][DFKQRVY]
NEK11	[DGKQ][ERS][DGMS][EFGV][CLS][LST][DET][S][GKPT][DFG][CLPS][GL][DFS][CKS][KLP]
NEK2	[ELMQRT][ADKRT][EIKLRSV][LNPQRS][FLNPY][ADEMNRTV][ADEGLRTVY][ST][DFKLPQRS][EILPQST][AGKLSY][EKMST][AEKPRST][IKLPQSTY][AKLRV]
NEK6	[ADEIKNPTV][ADEGILPQST][AFGIKLRSV][CDFGNQT][FLP][AGINPQS][EFKMNR

	SV][ST][AFGIMNPRVY][ADFGKLNPRTV][ADFHLNPQRST][ADGLPRSVY][ADFGKLNPRST][DGLRSTV][ADGHILNS]
NEK9	[EGHLS][EKPQ][AFLQTY][AGPSTV][AEGLMQ][ALMPST][EHQR][ST][IKLW][GMNTV][GHLTY][DPQT][DFPT][AHLPVY][EGKQSY]
NIK	[ADEKLQRT][AEGHKLQS][DEGIKLR][ADLSV][ADGKLNRS][CDEHLQRY][DEGKLT][STY][AFGLMQSTV][CDFKLRSVY][ADGLNQSTV][EHIPSTV][EFGLMQRST][ADEKQSVY][ADEGPSTVY]
NLK	[DILNPSTY][HMNPTY][AHIMQSTY][DEFNPSV][AEGHILRY][HIPRSTV][AFLMNV][ST][P][GILPQRT][GIMNSTV][DHKLNPTV][DEGLPQRY][AEPQSTV][CDGHILNS]
NME1	[AGLQS][ADLRSV][EGKLNSW][FIRSV][GIMNQRS][FHINQT][AEGIPR][DHS][ADEGNSV][ADGPST][ADGLSTV][ACDELS][AIKSV][AENT][EHNPQS]
NME2	[FQ][RV][GQ][RV][NR][IL][IK][H][GR][KS][DL][RS][EV][KQ][SV]
NPM-ALK	[DP][FGI][PSV][KST][GKR][DT][GIR][SY][IQW][DEK][LST][ENS][LRS][ILP][LSV]
NUAK1	[AFLNPST][GIKLS][LPTV][EGNRTV][GKPR][KPST][ADGLNV][S][DFQSY][EGNPRS][ANRTY][FLPV][ADELS][DEFGS][CILNRS]
OSR1	[AEHKLMRSTY][INPQRSY][DEHSTY][DFHPRSTY][CFGHNSY][AEHLMRTY][DGHLNQR][ST][AFGHIMTY][ADGHQSTY][AGHILNPQSVY][GLNPQRSTV][EFGKPTY][ACDEFIKMPRTVY][ADGIKLMNRY]
P38A	[AGIPQST][DEGLPST][AGLPSTV][DEGLPQST][AGLPST][LPST][AGLPQT][ST][P][DEGLPSTV][AEGLPRS][GKLPSTV][ADEGLPRS][ELPQRS][AGLNSVY]
P38B	[DILPQRST][ACDELPST][DELPQS][FILS][AEGILQS][DHLPS][ALMST][ST][P][ELPSTV][ADEHNS][EFGPQST][AEGLRTV][ADHLSV][DEHILSY]
P38D	[GQRST][ADGPRSTV][EFKPRS][ELPRS][FLPRST][ILNPRST][LRSTV][ST][NP][EPRSTV][DGSTV][AKLPRS][EGPSTV][ADNPRST][ILPSTVY]
P38G	[FGLPQTV][ADFGKLNPTVW][ADEGPQSTVY][DHILMPQRS][DGLPQSTV][ADHLMPSTY][AEIKMPSTVY][ST][P][AGILPRSTV][DEHKPQRSVY][ALNPQRSTVY][AGINPQRSTY][ADEGNPQRS][AFGINRSTVY]
P70S6K	[GILNPRS][EGKRS][AKRS][IPRSV][R][LRST][AKLRST][ST][AEFLPST][AGPRST][AEFKPRS][ALPQSV][ACPST][AGPST][AKPQST]
P70S6KB	[AINPRS][AGKPRS][KLQRVY][IPRSV][R][ALQRST][LST][ST][EFGLRS][AGLPQRS][AEGLRSV][AGPSV][CDEMPST][FGIKLST][KLNPRSV]
P90RSK	[AEGLPRST][AGKPRST][DLRS][KRS][R][KLRST][DGLPS][ST][ADEGLSTVY][AGPS][ADEGKLNS][AEGIS][AEGLPST][EGKPST][ADEPS]
PAK1	[AEKPQRST][AEKPRT][DLPQRS][AEGLRST][KLR][PR][DKLRS][ST][ALMPSVY][AEGLPSVY][ADGILPSTY][AEGKLPS][ADELRST][AEGPSV][AELRSV]
PAK2	[ADEPR][AEGKLPRT][CIKPST][DEKLPRSTV][AGKR][KPRT][ADIKLMRS][ST][AFHILMS][FIKLSTVY][ADEGINPV][DEFIKLPQT][EKMNPST][EGKLSTY][ADGLNQW]
PAK3	[EGNPRTV][AGKLST][GKLPTV][EGLRSV][GKNPR][HIKPQRV][ADGKLNS][ST][GMQRSV][AISVWY][DEGKNP][AEGKMV][DGMPSW][GLNS][GKRTV]
PAK4	[ADEIKPQR][DEKLQRV][DFIKPRVY][AEKNPQSV][KLRS][AEFKMR][AKLNQRTY][ST][EGILNRV][AEPQRSV][ADFGLRS][AFGHNRSTY][ADEKLMPRS][AFKLMRSTY][ADIKLMWY]
PAK5	[EHLNS][GNRST][DKLRS][AGIPS][AKRV][HPSY][ADNS][S][AILPY][AKLPY][AGL][AGRT][GPQST][DEFT][EFGL]
PAK6	[HPRSY][DGLQ][ARV][GKLS][DKRT][CHPR][GKSY][ST][CDSV][KSV][GIRSV][FNRSY][DFLPR][AKRSY][FGIRW]
PASK	[AELY][EFRV][GNRY][DKPT][FLMR][CFPRT][AGQRY][ST][AFIV][ACEPY][GPTVY][CGRST][AIPV][EIPQS][EKLY]

PBK	[KLQTV][FGKN][GKLQS][DKLT][AIKL][LRSY][AKLT][ST][DNPT][DEGSY][GKLSV][EGKT][AKLR][LPVY][IKLRV]
PDGFRA	[IPR][DLSV][DGIPS][ADEHS][DEG][HNSY][AEGI][Y][ITV][ADGIY][PRV][DLMQT][GPQY][ADKLM][IKNPQ]
PDGFRB	[ADEILPQST][DGIKLMRS][DEIKLMNPR][EGKPRSVY][DGKPSV][ADGHKNPSV][ADEGIKNRT][Y][DEGILMTV][ADGINPSVY][IKLMPRSV][AEGLQSVW][DEKPQS][DMNPRST][DEIKLPRSY]
PDHK1	[NST][LMRY][RSY][DGY][HMP][G][HTV][S][MTVY][ERS][DRT][APR][AEG][AEV][IS]
PDHK2	[NST][MRY][RSY][DGY][HMP][G][HTV][S][MVY][ERS][DRT][APR][AEG][AEV][IS]
PDHK3	[ST][MY][RS][DY][HP][G][HV][S][MY][RS][DT][PR][EG][EV][IS]
PDHK4	[ST][MY][RS][DY][HP][G][HV][S][MY][RS][DT][PR][EG][EV][IS]
PDK1	[AEGHLNRST][DEHIPQSY][EGKLNSY][ADFGKST][LPRST][AGMQST][FHKNST][ST][FLPY][CSV][AGV][PT][APTV][ELNSTV][FSY]
PERK	[IMNQ][CEIK][GLQV][DLMT][DEIS][CELS][KLN][SY][ADER][ILRS][KRSV][IRS][AIR][DRS][ILV]
PHKA1	[KST][EGN][FIV][GMS][PQV][AEL][IKR][S][EIV][HIR][EKP][IQT][DGV][AES][FNV]
PHKB	[AHT][GRS][KLT][KMV][AKR][ERS][GQV][S][SVW][KTY][ESV][LNP][AEL][KPR][ERS]
PHKG1	[AEKMNQST][AEGIKMNPV][AFGIKLTV][AGKMRS][AIKNPQRV][AEGILQTY][AGIKLMRSV][S][DEFIMSTVY][EGHILRSV][AEGKNPSV][HIKLNQRTV][ADEGIKMPRVY][ACDEGHST][FILNQRSV]
PHKG2	[GY][LP][LS][GM][R][R][AH][ST][DV][G][GV][A][DN][IP][QR]
PIK3CA	[EHNPS][DNQRS][AEGRTV][EFLRT][DELRV][GKQST][FHIVY][STY][EFLSY][ACETV][AEGLV][DEKPT][DIKNS][AEKTV][DLQY]
PIK3CD	[LNR][KWY][KLV][ALV][DHV][ADN][ELV][SY][DKT][DSY][MNS][PRV][GMQ][NS][AK]
PIK3CG	[FILS][AFKPV][AGNPQ][AGHS][AEFPQ][KNQT][HPSV][S][ADK][KLPT][AELV][GLST][AENS][DEPS][DGNQ]
PIM1	[AEGKPRS][GIKPRST][KLQR][GKLRS][GKR][AHKLPRS][EIKLPRS][ST][DELSVY][DGLPQST][AEFGKLPST][DEGKLNPST][KLRST][EFLPQRST][EFKLPRSY]
PIM2	[AEGPQRW][ACFGKLPRT][EKLPRT][AEGIKLRS][IKPRT][AHKLQRS][DGMPQRSTV][ST][ADEGMRSWY][EGLMNPRT][ADFLPSTV][DEFGLNQS][EFGKLQSTY][AEGHQRSTWY][AEFGHIPQRS]
PIM3	[EGHKPQ][CFGIQRT][ELPQR][AGKLPRS][ARS][AHPRST][AGKMNRS][ST][ADEHNY][EGHPST][AFGHPQT][AEGHMNS][AGLRST][EGQSTW][ADEFMPS]
PINK1	[ADGMN][CGKLWY][DENQ][ILY][DEGKPQS][AFKNQ][EGIQSV][ST][EISTWY][LRSVY][EFHKV][ADILSY][IKRVY][AGLQR][GNQR]
PKACA	[AEGKPRS][AGKPRS][AKLPRS][AEGKLQRST][KR][KRS][AGKLPRS][ST][AFILSV][AEGLPSV][AEGLPS][AELPRSV][AEGLPRSV][EGLPRSV][AEGKLPRS]
PKACB	[PS][EM][GS][EP][K][HL][HT][ST][DP][FS][GS][IV][S][DV][DI]
PKCA	[AGKQRS][AGKLPRS][KLPRS][AGKLRST][KRS][KQRS][AGKLPRS][ST][FKLRSV][KRS][AEGKRS][AKPRS][AKLRS][EKLPRS][AEGKLPRS]
PKCB	[KLPQRS][ADEGPRSV][EGHKLQRS][AEFGPRSV][FKRSTV][AEGKQRST][AFGKRST][ST][FKLRSV][AKLRST][AEGKNRS][KPRS][EGKLRST][ADEKLRS][AGKLNPSVY]
PKCB ISO2	[ALPQRV][AGKLPRV][AKLPSV][AKPRV][EKRT][KQRSV][AKPRS][ST][AFKQRS][IKLR][KLNRV][IKLRSY][AKLQRT][HKNQR][AKLNPR]

PKCD	[AEGPRST][ADEGKLPRV][GKLPRSV][AFGIKLPR][FKLR][KPRS][AGLPQRS][ST][FILPQSV][AKRS][AKLNRSV][KLRSV][ADEKLPRS][AKLPRSV][AGIKLRS]
PKCE	[AGKLNPQRS][AEKPRSV][IKLRSV][DGIKRS][KRST][AGKPRS][AGKLPRS][ST][FGKRSTVY][KRV][GKNRS][GLRSTV][KLPQRSTV][ADKLNPRSV][ADEGKLRSTY]
PKCG	[AIKLMPQST][GKRSTV][AFGIKLNPRST][AGKNPRSV][AEGKQRSTV][KRTV][AGKPS][ST][FKLS][KRT][EGKNR][KLPRS][KLQRST][EGHKRS][EGKNPS]
PKCH	[AGSV][AEGLR][IKLQR][AIPS][FGKQR][ERT][KRST][ST][AFLTV][AKRV][EGRV][KPSTY][CGLPV][AGHKNV][GKPVY]
PKCI	[CDEGKLPQRV][EFGIKRV][KLQRTV][DGLPS][GKLRST][ACGHKRSVY][CEGKMQRS][ST][AFKLPQ][EHIKPR][ADFGMNQRV][GIKNRSVY][GKLPQRT][DEHKLQRST][CEGKNRSVW]
PKCT	[AGLPQRT][CGKRSY][GKLQRS][ADEKLRS][FKRS][DHKPRY][GKPS][ST][FGILPQ][KRTV][AFKLQRS][AEIKLR][AEKLRS][AELQRSTW][AFGIKST]
PKCZ	[ADGKPRS][AGKLPRSVY][GKLPST][AGKLPRST][GKLRST][ADKLPQRS][AGKLPRT][ST][DFKLPR][DKLRS][ADEGKLRS][AEKQRS][AGLQRST][ADEKLRSV][DEIKLPRS]
PKDCC	[AF][QS][GY][HV][DG][LS][G][SY][CT][KV][AR][EV][FY][GR][AV]
PKG1	[AGKPRS][AGKNRST][KPRST][AEGKLRST][KR][KR][AILPRSV][ST][AFPQRSTV][AEKLQS][EGLNPR][AEFGQSV][DIKLPSTV][EGLPRSY][AEKLPRSV]
PKG1 ISO2	[AEFMPRST][ACEHPQST][EFKLMPQSY][AELMRSTY][QR][AGKNQR][DILPQTVY][ST][AEGLNQRSTW][ADEGMPQS][GILPQTV][FGKLQST][AEGPQRSV][DFGIPQY][ADEPRSVW]
PKG2	[IKLMPQRSTV][DEGIKQRS][AIKLQRTV][EHIKLRSV][AGKRS][GKLR][EFGKLMNTV][ST][AFGILQSV][GHLRST][EGKLSV][AEGPRSV][EFKLRS][ADGHLPQR][AEGLNQRST]
PKM2	[DEK][GKPR][GRT][ESTV][AE][ARV][EPRS][SY][DLS][DEK][EPT][EKNS][FGNP][EGIP][CPV]
PKN1	[FGKMP][ADFKLNSY][FGKLRT][ADEKLNSV][AKRTV][AGKNRSTV][FGKLPQST][ST][EFKLP][CIKLS][GKLQRSTY][ALPRST][AGLPQRST][ADEFKLPQRS][AEFLMNPS]
PKN3	[AR][HS][IL][KV][R][KS][PR][ST][AL][RS][RS][L][LS][QS][EM]
PKR	[DEIMNRTV][EIKLQRV][GKLPQST][DEIKLRS][AGIKLMST][AEGIKL][DEGLRT][STY][ELPRTV][ADLRS][IKLNRS][EILMRS][DFKRSTV][EGHKLPRS][EGILQR]
PLK1	[AGKLPQST][ADEKLST][DELPRS][AEKLPS][AEGKLRT][DELNS][DEKLS][ST][FLPSV][DEILSV][DEGKLS][DEGLPST][ADEGLPS][AELSTV][DELST]
PLK2	[DEIKLNSV][ADEGKLMQRSV][AEFGHIKLPRS][ACEGLNPSTVY][ADEFMNRSTV][ADEGHILMNPQR][DEGHIKPQRS][ST][EFLMPSY][DELQSVW][DEGKLMQRSVY][DEFIKLNPSY][ADEFHIMPQST][DEKLPQSY][AEFHIKLNPQRY]
PLK3	[ADEIKLRSTV][ADEGMSTV][ADEGISTV][ADFKLPQSTVY][ADEKLMPQT][ADELMST][ADEFKPQSTVY][ST][ADEFKLMPR][DELNST][DEGLQRVWY][DEFGIKLMPS][ADELPQRST][ADEFHKLNPQS][AEKLNPSY]
PLK4	[ADGILST][GISTV][AFGHLST][AEGST][GIKLRST][HNST][ADGLST][ST][FGILST][ALST][ADGILST][AEGKLST][ILRS][AGLRS][AEGLST]
PRKD1	[AEGKPRS][AFKPST][LV][AKRSV][KR][AHQRST][AKLRSV][ST][AEFILNSV][ALNPRSV][AGLNRSY][ADEGLPRSTVY][AGKLPRSV][AELPSY][AKLPRSTY]
PRKD2	[EGHKQRST][ADFGHKLNPRST][IKLV][ACEKLPQRST][EKR][EKMPRSTV][AFHIKLMQSV][ST][AIKLNQSTV][ADGLNPQRST][EGKLPQSY][AEFGIKLNPRTV][AEGHKLPQTV][ADNPRST][AEGLPRSTV]
PRKD3	[AGR][EPRS][IKL][GIS][FGR][EHRT][IKQR][S][FISV][PRV][GKLR][HPST][LPQV][A

	RSV][GHPY]
PRKX	[ER][KP][LV][P][PS][GR][SY][S][IR][GK][SV][FK][DV][FS][EP]
PYK2	[CFPRSV][DGKLNS][DEIKLPQS][DEGKQV][DEGRS][DEGHPR][AEILP][Y][APSTV][AEHMTY][FIPSV][CPRST][ADEGK][EFLNPQ][AGHKRST]
QIK	[AELQS][AGHLNT][AHLQR][HKLNQRSTY][FHKLRSW][CGST][GHLNRST][S][CDHLNPS][AGLPS][ALNRSY][AILV][AHLQRS][APQTVY][AEFLST]
QSK	[LS][AGLT][LQR][LNQY][FHRS][GST][GLNR][S][DLN][PS][ANS][ILV][HQS][AQT][LST]
RAF1	[AEGLPRV][IKPRS][DGIQR][GLNQS][LMQR][AHIRSV][ADLNRS][ST][EFMRTY][APRV][AGNSVY][GLSTV][DFHLRT][ELRSV][ADEGVY]
RET	[ADLMPRT][GMNRVWY][GISTV][DEFKNS][DELMNS][DEKLPRST][DEGLSV][TY][AEGLV][ADEKLMN][DLMSTV][ADGRS][AGKPSVWY][GLNPSY][ALPRTW]
RET ISO3	[DT][PW][GI][ES][AN][AK][LP][Y][GL][KR][IT][KS][FH][AI][CF]
RIPK1	[IKLNP][ANSV][DIPSV][ADFIKL][FGKMR][KLMS][AEKSW][S][ADFKSY][DEFKLS][EFLMN][ADELNW][ENS][AEGKS][AEGHLP]
RIPK2	[DK][LW][IR][M][KM][ES][DL][SY][EQ][LS][RV][S][ST][K][PS]
RIPK3	[EFKST][EKLMQ][ILMNRST][CHKQRS][IKNRT][LQST][EINPQRT][ST][AIMPRSV][DKMPST][ACLRS][GKLPT][AGKLST][AKT][EFKRST]
ROCK1	[AKLPQRS][AGKRS][AEKPQRS][DGKPQRSV][KQRS][KRY][AGKRST][ST][GLPQRSV][GKNRSTV][AFGPQSV][AFGMST][AGLPRSV][AFGKPQTY][DEGLPQRS]
ROCK2	[ADKLQRY][GKPR][PQRS][DFGPS][KRST][KRY][AHKRST][ST][LQSV][GIKRSV][AQSVY][DEISTV][AFLMPQRSV][ADIKPQTVWY][DEFGIPTVY]
RON	[DELQRSY][DMTVY][ACFHMRY][CDGIPVY][ADGKN][AEKMPS][EHLPTY][Y][HLPSVY][AHNSV][EHIMV][DHKLNQ][ACKNPR][CFKTV][ADGTVWY]
RSK2	[APRST][AFGIKRT][IKLPRT][GKRST][ARS][KLNRST][GKLPQRS][ST][FLPRTV][GPRS][ADEGRS][DEGKS][AEGLPST][GKLPRS][DEGKRS]
RSK3	[ADEQ][GHIK][DKLN][GKSV][ELR][ALQR][LMPY][ST][FPRY][CER][GKLY][IST][AILT][ELN][DFRY]
SGK1	[EGILPRST][GIPQRST][LR][AKLMPS][R][LRST][HLNPRSY][ST][AEFGIKLSVY][DEGLPSTV][DEGKLRST][AGILPRSTV][AEGLPQRSTV][AGLNRSTV][DEGLRS]
SGK3	[DGLRS][GHLW][EFR][KPRT][R][HKT][ADT][ST][AFNVW][AGQS][DER][DPSV][CLQTY][AEFKL][EGKPV]
SIK	[AKLPS][AGLSTY][LMQRS][EKLNQSTY][DFGHRSTW][CEGLST][GLMNRSV][ST][ADLNPT][ACPST][ADGNPSY][AIKLPSV][AGHPQST][AGHNPQT][EGLSTV]
SKMLCK	[AKNPT][AEKQ][AFPQV][AHLQ][EGHQR][DGRS][GPRST][ST][CNRS][GNPSV][ADFIV][EFIS][FIMSV][AEFMQS][ACEFST]
SLK	[S][S][P][Q][R][V][T][S][S][Q][Q][Q][T][R][I]
SMG1	[AGHPQS][EGLMQS][GILSV][DELQV][GPTV][AEGP][DL][ST][Q][DEGPY][EST][EFLTY][FLSY][DEGQ][DFGL]
SMMLCK	[AGKRS][AGKR][AKMPRV][GKPQ][AGPQRS][AGKQRS][AKNST][ST][AGNRS][ALNRTV][FLRV][AEFKSV][AELMPRS][AFGIKM][DEFISTV]
SRC	[DEKLPRS][DEGKLPSV][ADEGLPS][DEGNS][DEGQS][DEGNPS][DEILPSTV][Y][ADEGQS][AENSTV][FILPSV][DEGPRSV][AEGKPST][AEGKLPS][DEGKLPRS]
SRPK1	[RS][GS][NPRY][GS][NR][KRS][RY][S][PR][ES][PR][RS][ER][RS][PRS]
STLK3	[AELMSTY][ANQRSY][DHNT][FKSTY][GHSVY][HLNQRTY][FLMNR][ST][FHIMPVWY][DGTVY][AGHKLRS][DENTV][KPT][CFMPY][DEGIM]
SYK	[DELST][DEGLPST][DEMPV][ADEGST][DENS][DEGNPS][DENPSY][SY][AEKLV][ADEKNPST][ALPQSV][ADENPQTY][AEGIP][DEGIKPSV][ADEGKNPQS]

TAF1	[DLV][DENQ][DEIS][EK][DLPQ][DLNW][EFNV][ST][DE][DE][DEPQ][GHV][PQS][DE][ELN]
TAK1	[CFHILST][DMPRTV][GILST][CHNQRS][DKNRST][AHKST][AEGMQS][ST][AEHNSV][ACDLNPS][AGKNSW][DGLMPS][AIKLPS][ADKLNPT][AEGKNPR]
TBK1	[AEILPQSV][DELQRS][DEGHLPRS][AEGKLRS][DGLPQS][AEFLQS][GILNPSV][ST][FILMS][ACDEGPS][AELNSY][ADELPSTV][DLPQRSV][AELMPRST][AGKLPRSVY]
TEC	[AEGKLRST][AGHKLQRSTV][AEGHKLNPQTV][ACEGLMRSTVY][AEFGILSV][ADEMNPQS][EGHLNQRTWY][Y][ADLNQRSVW][AFIKMNRSTVY][DGILMNQTVY][AEFGKPQRSTY][DEHKMNPQRSY][DKLNPQRT][ACDGIKLMPQV]
TESK1	[E][P][L][A][V][MV][AG][S][GP][VY][AQW][MV][AS][DP][EG]
TGFBR1	[EGHQST][EFMPSTV][DHINPRST][ACFGHLSV][DEGLRSV][ACEIRST][CDGLMNPQS][S][EMPST][AGMPQS][ACILPSV][AHNPST][CGIPST][CDILNPS][AGNST]
TGFBR2	[ADGKLMSTVY][ADGIKLPQRTV][ADGIKLMNRTVY][ADEFGILNSTY][ADEGLMPTY][ADEMPQST][AEFGLMNPRSTV][STY][EGKLMSTV][ADEGHLQST][CDGKPRSV][AEGHLPS][AFGIKLPSTV][AGILNQSTV][EGHLNPQRS]
TGM2	[AT][KT][KQ][AT][A][R][K][S][AT][GP][AG][KT][AG][GP][RV]
TIE2	[LMT][LSY][ERV][EGN][QRT][EKT][LTV][Y][EV][KN][FKT][T][LMY][AGY][EGR]
TLK1	[ETV][KLP][PQS][ST][AIV][PRS][GKL][ST][PT][GP][GP][KQ][AKP][FP][KR]
TLK2	[GP][GW][LS][LT][PS][E][N][S][LM][DN][CV][IM][L][E][S]
TNNI3K	[AR][GK][K][FK][KS][KR][IP][ST][AL][RS][R][KV][LR][IQ][LS]
TRKA	[ADFHR][ADGI][GIMY][DESY][DNPQST][DPT][DIQTVY][Y][FQRSVY][DNRSTV][DGPV][AGQR][CGRY][KLRTV][DHMT]
TRKB	[DEFPRV][ADGQV][GIKMS][ADES][AEINQS][DPT][CIQTVY][Y][FQSVY][GLNQST][GINPSV][GKTY][CGHNPS][GIKRS][ILMPQR]
TSSK4	[CES][IL][ELS][HIS][LRT][PRS][PQ][ST][SY][CER][GK][ILS][FLV][ANS][DEVY]
TTBK1	[GKPSV][ADGNSV][DEGIKPRSVY][DGIPRST][GMPQRSV][DGLPSTVY][ADGKRSTY][STY][GILPS][ADGPQRSVY][ADGHLMPSTV][AEGLPRSV][DGKPSTV][DGLNPRST][AGPRSTV]
TTBK2	[DEGQS][DEGRSY][AESV][EGQRSY][DGQRS][ADEGNRS][AGRSY][ST][DERSVY][ADFGRS][ADEHRST][DEFITY][EGS][DEGHPR][DEFHV]
TTK	[ADEGKLQSTV][EGKLNPQST][EKLQSTV][AEGILNSV][DEKQRST][ADEKLNPSTV][GKLNQST][ST][DEIKLPSTV][ADELNPSV][AIKLSV][ADIKLMSV][ADKLRSV][AEGKNPQST][ADEKLRSV]
TXK	[KR][IY][QV][LV][DK][AD][EL][Y][DV][FS][LS][FP][GR][AE][KP]
TYK2	[ACHKLNPSV][ADEFGHKPSVW][ACDEGIKLPQS][CEGKNPRS][ACGHIKSWY][ADEHILPQTY][AEGIKMNPY][Y][AEFHLQRSVY][ADFKNRSVY][EFGHNQRTV][CELNPQRSTV][CDEFGKNPSTW][CDEGLPRSTV][DEGKLMPRS]
UL97	[DIKLMPSTV][AGINPSTY][AFGILPRSTVY][DEHIKNPS][FGIMPT][KLPQTVY][HIKLMPRS][ST][P][IKLRTVY][KPRS][FHIKMRST][GINPSTV][EGKLMPSY][DGKNRSTV]
ULK1	[DELQT][AEKLRST][AILMQS][DNPST][ILMRV][ADGQS][AGKPSV][ST][DFLSV][DELPST][ADGLPV][CDLPQST][AGHLPRV][DPQRSV][AGHLPSV]
ULK2	[ELST][AHLS][AQRS][PRT][IMR][NQV][FSV][S][FLMV][PRSV][CLN][LQST][DNQR][CDST][AHIS]
ULK3	[DEKL][AKL][ADEK][AGLQ][EIL][GSV][ADES][S][ASV][ANR][AMQS][ALS][CKLS][ELT][ELRY]
VEGFR1	[ACDEGV][CGQSTY][NQRS][DMNPS][DFGPV][DGKPV][DFLR][Y][INQSV][EGNPS

	T][ADIPV][FNPSV][AIKLPS][FIMTY][LMPS]
VEGFR2	[ACDEFGHLRV][CEFGHILMPQS][ADILMQRSY][ADKLNPQT][ADEGKPR][ADFKPRT][CDHILRTY][Y][DEIKNVY][ACDGMNRV][ACEFKLPSTV][ADGILMPSV][DFGHIMPSTY][AFILNPSV][DEGPQRS]
VRK1	[AEFGKNTV][DEGKLNP][EKLPQS][ADLT][AKLMPRS][ALMNQRT][ADEIKMPRT][ST][FKPQRSTV][DEFGIKQS][DGHKLQRTV][AEGHKLR][ADEHKLNRW][CDEFKLPQR][CDEFIKLRSTV]
VRK2	[ENPQ][EKLP][ELPY][ALRV][EGKMS][IMQRST][EKMPT][ST][FLPQRST][KPQSV][DGHKPQT][DFHKLRT][ADEHLRSW][DFKQR][ACDFHLV]
WEE1	[ESV][EKL][IK][GI][EGN][EGT][FGT][TY][GSY][GNV][KV][EVY][IKY][AFGK][ALR]
WNK1	[EKLSTVY][AGQRT][AEKNRSTY][KLNSTV][AFHKPVY][AEGHRT][AGKMRS][ST][EFGLVW][AHILMNRTV][GKLMPR][ADEHST][FKPQVW][CEINRSTY][DEFGMSW]
WNK2	[IKV][ER][AKR][LSV][FLP][AGT][KRS][ST][GPV][DHI][GIL][AHT][KPQ][ERT][AEF]
WNK3	[IKLSTY][AENQR][ADETY][FKLSTY][FGSVY][AEHLQY][KMNQR][ST][FILMVW][DGIMT][AGHKMV][DENTV][HKPQT][AEKMRSY][DFIMQSY]
WNK4	[PRSTV][GHRS][ILNRY][KMPTV][HKPRV][GRS][KNS][ST][FGLS][HKSVY][EGLM][HRSTY][KPSTV][CFT][EGW]
YES	[ADGHIKMPRTV][EGIKLMNPRST][ADEFGILPST][AEGLNPS][ADENPQV][EFGHKMNS][AEFGHIKTVY][Y][EGKQRTVY][AGKNPQRSTVW][EFILPQRV][EGHKPQRTWY][AEGKNPRV][AEGKLPRT][ADHKLPRSV]
YSK1	[EGV][LNR][EGL][IRS][AIV][ANT][DLQ][ST][AFL][AGR][AQR][AHT][FLR][IR][AK]
ZAK	[AHS][NRST][FHKLR][AEFHT][AHNT][HNRV][HKMST][ST][AHLQT][EHMPV][AGLMS][LSTY][FGLSV][GIPV][GPTVW]
ZAP70	[DES][DEGLPS][ADEPT][DEG][ADEPS][ADEPRS][DGPQY][Y][DELTVY][EMNPQS][LPSV][FNPQRS][EPQS][DEKLS][EKPQS]
